# Anion Intercalation into Graphite Drives Surface Wetting

**DOI:** 10.1021/jacs.2c13630

**Published:** 2023-03-28

**Authors:** Athanasios A. Papaderakis, Andinet Ejigu, Jing Yang, Amr Elgendy, Boya Radha, Ashok Keerthi, Anne Juel, Robert A. W. Dryfe

**Affiliations:** † Department of Chemistry, University of Manchester, Oxford Road, Manchester M13 9PL, U. K.; ‡ Henry Royce Institute, University of Manchester, Oxford Road, Manchester M13 9PL, U. K.; § Department of Physics and Astronomy, University of Manchester, Oxford Road, Manchester M13 9PL, U. K.; ∥ National Graphene Institute, University of Manchester, Oxford Road, Manchester M13 9PL, U. K.; ⊥ Egyptian Petroleum Research Institute, 11727 Cairo, Egypt

## Abstract

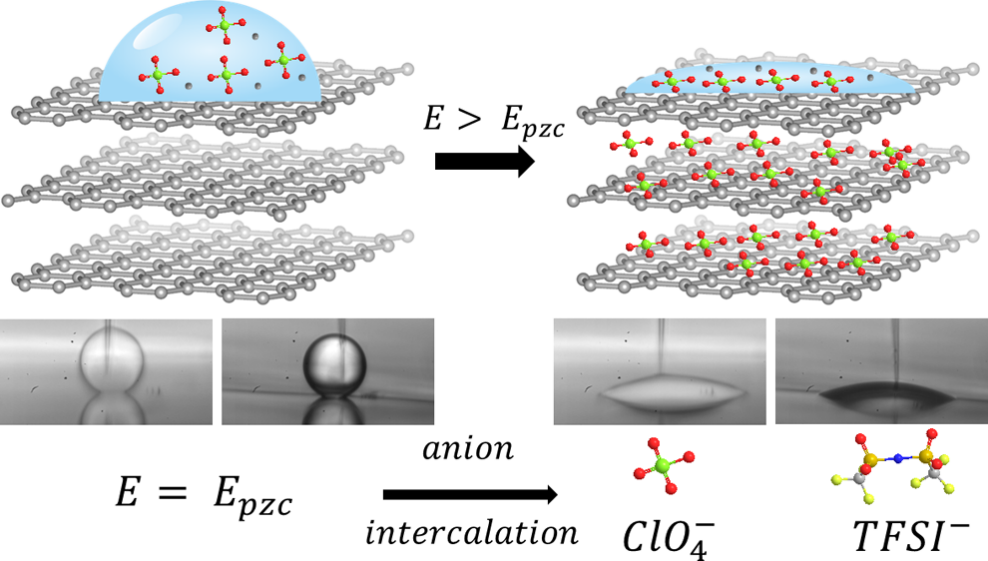

The unique layered structure of graphite with its tunable interlayer distance establishes almost ideal conditions for the accommodation of ions into its structure. The smooth and chemically inert nature of the graphite surface also means that it is an ideal substrate for electrowetting. Here, we combine these two unique properties of this material by demonstrating the significant effect of anion intercalation on the electrowetting response of graphitic surfaces in contact with concentrated aqueous and organic electrolytes as well as ionic liquids. The structural changes during intercalation/deintercalation were probed using in situ Raman spectroscopy, and the results were used to provide insights into the influence of intercalation staging on the rate and reversibility of electrowetting. We show, by tuning the size of the intercalant and the stage of intercalation, that a fully reversible electrowetting response can be attained. The approach is extended to the development of biphasic (oil/water) systems that exhibit a fully reproducible electrowetting response with a near-zero voltage threshold and unprecedented contact angle variations of more than 120° within a potential window of less than 2 V.

## Introduction

Wetting phenomena are ubiquitous, underpinning physicochemical processes occurring in both nature and artificial systems. Even their simple macroscopic observation can provide significant molecular insights into the properties of interfaces as well as those of the adjoining bulk phases.^1−3^ A number of diverse technologies, from electrochemical energy conversion,^4^ storage^5,6^ and capacitive deionization (CDI)^7,8^ to variable optics,^9−11^ displays,^12,13^ and lab-on-a-chip systems,^14,15^ are driven by wetting processes which occur under the application of an external electric field, a phenomenon referred to as electrowetting.^16^ Controlling wettability under these conditions is of paramount importance for product design, since any variations in wettability can have significant implications for the operation of the devices (such as lack of adhesion,^17,18^ hysteresis,^19−21^ and loss of electrochemical activity^22−24^), resulting in performance decrease and/or failure.

For the range of aforementioned applications, carbon-based materials play a dominant role due to their notable physicochemical properties, including high conductivity, (electro)chemical stability, and unique structural characteristics (e.g., layered structures in the case of graphitic materials).^25^ In particular, allotropes and/or composites of carbon are established electrode materials in alkali metal ion batteries,^25,26^ supercapacitors,^27−29^ fuel cells,^27,30^ electrolyzers,^27,31^ and CDI devices.^32,33^ In stark contrast to the role the carbon/electrolyte interface plays in electrochemical systems, wetting applications in electronics are normally performed with a dielectric layer between the conducting substrate and the liquid phase (termed electrowetting on dielectric, EWOD),^16,19^ thereby decoupling the substrate from the electrowetting response. The presence of such an insulating layer prevents substrate and electrolyte degradation, but it renders the use of high voltages (usually a few tens of volts, or exceeding 100 V, depending on the thickness of the dielectric^16,34^) necessary to induce significant changes in the contact angle.

**Figure 1:**
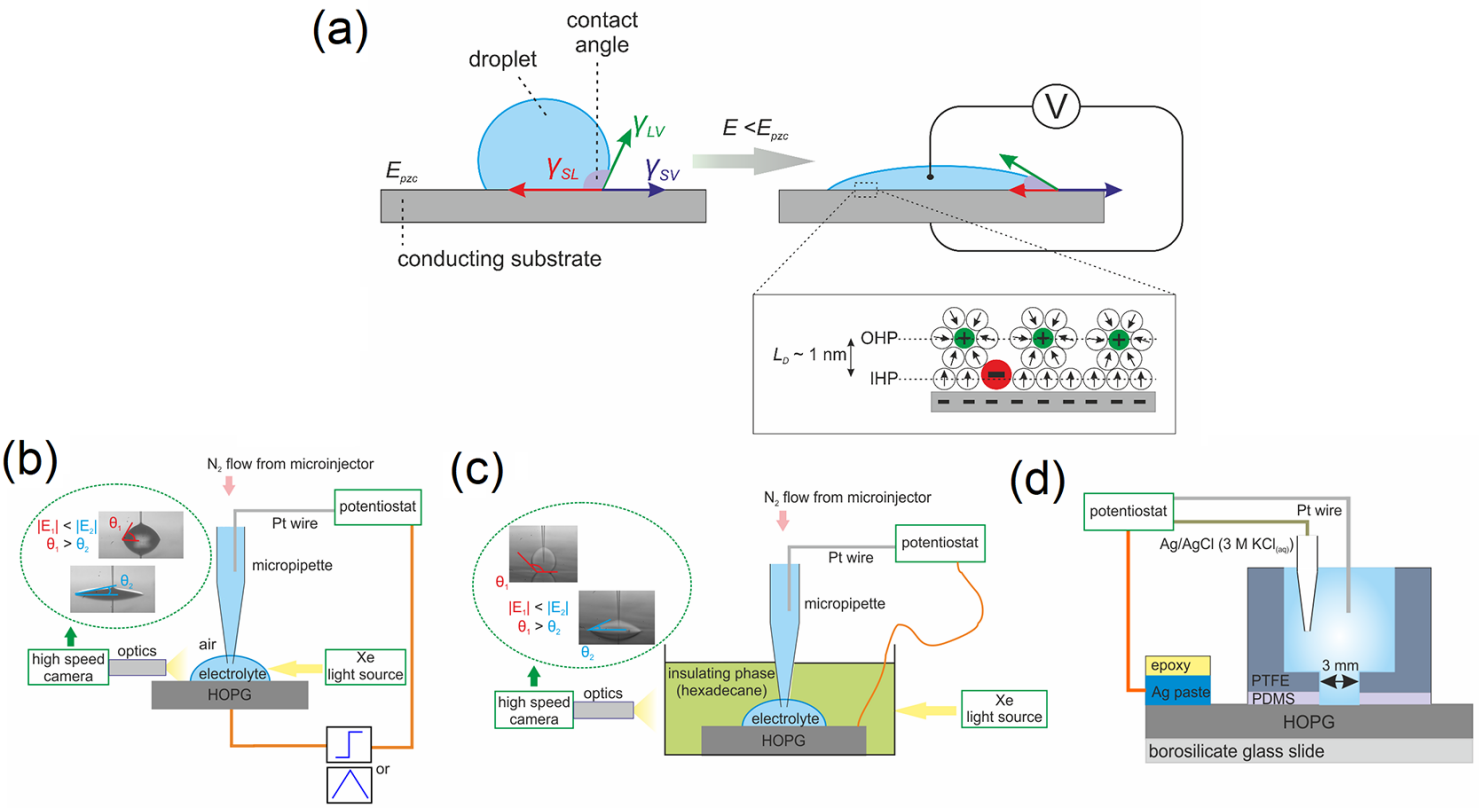
(a) Illustration of electrowetting directly on conductors (EWOC) in air at an ideally polarizable interface. At the potential of zero charge, *E*_pzc_, the interfacial tension at the electrode–electrolyte interface, γ_SL_, is larger (per unit length) than that at the electrode–air interface, γ_SV_. Application of a potential bias away from *E*_pzc_ (e.g., more negative) restructures the EDL and decreases the electrode–electrolyte interfacial tension causing the droplet to spread. γ_LV_ and γ_SL_ represent the liquid|vapor (here air) and solid–vapor interfacial tensions, respectively. Inset: Illustration of the inner (IHP) and outer (OHP) Helmholtz planes of the EDL, in the presence of specifically adsorbed anions. The thickness of the Helmholtz layer is approximated by the Debye length of the electrolyte, *L*_D_. The content of this figure has been adapted from ref (35). Schematic representations of the setup used for (a) liquid–air electrowetting experiments, (b) liquid–liquid electrowetting experiments, and (c) EIS measurements. For a detailed description, refer to the relevant parts in the Experimental Section in the Supporting Information.

EWOD is in fact a more recent development in electrowetting which, in its original incarnation, was performed directly on conductors (electrowetting on conductors, EWOC).^35^ The first experimental evidence of electrowetting directly on conducting substrates was provided by Frumkin, who, following the predictions of Lippmann’s theory of electrocapillarity, demonstrated the striking effect of potential bias on the shape of an oil droplet in contact with a mercury electrode immersed in an aqueous electrolyte.^36^ The overall mechanism of the phenomenon for ideally polarizable interfaces (i.e., no faradaic reactions occur) is based on the decrease in the potential dependent solid–liquid interfacial surface tension upon application of a bias away from the potential of zero charge, *E*_pzc_, i.e., the potential at which the net charge at the electrochemical double layer (EDL) is zero^37^ (see Figure 1a).^35^ More recently, in an attempt to decrease the energy demands presented in EWOD devices, Kornyshev and co-workers introduced, in a series of seminal studies, an alternative EWOC route based on immiscible electrolyte solutions.^38−41^ In these studies, a sputtered gold film electrode was used, although significant wetting hysteresis was noted, resulting from its micron-scale roughness. The authors subsequently alleviated the observed hysteresis by implementing a voltage pulsing technique.^41,42^ Finally, most recently, our laboratory has demonstrated that graphite is a promising material, exhibiting ultra-low voltage electrowetting on its basal plane, without the use of an insulating coating in both air–liquid and liquid–liquid configurations.^43,44^ In the latter case, despite the amplified electrowetting response, the presence of an ultra-thin insulating layer between graphite and the aqueous electrolyte results in significant threshold voltages. However, we have very recently shown that by using highly concentrated aqueous electrolytes on graphite, a response that is fully reversible, reproducible and symmetric relative to *E*_pzc_ can be attained within a potential window of up to 2.8 V.^45^ By extending the approach to the liquid–liquid configuration, the threshold voltage is decreased, though not eliminated. Overall, it becomes evident that the study of electrowetting on carbon-based materials is of great significance from both fundamental and applications point of view.

The aim of our work is to utilize the unique physicochemical and morphological properties of graphite to drive and effectively control surface wetting. We investigate the electrowetting behavior of the basal plane of highly oriented pyrolytic graphite (HOPG), as a well-defined carbon system, in the presence of electrochemical processes that alter its intrinsic properties. On this basis, we study the effect of ion intercalation into graphite by electrolytes commonly used in electrochemical energy storage devices^46^ on its wettability. Remarkably, we find that intercalation-assisted wetting on graphite is a quite general phenomenon. The manuscript is organized in terms of the electrolyte systems we investigate. We describe our initial findings on single-phase (electrolyte) wetting, i.e., with air as the surrounding medium, with lithium perchlorate (LiClO_4_) in propylene carbonate (PC). The study then extends to highly concentrated electrolytes with anions capable of intercalation, namely, lithium bis(trifluoromethanesulfonyl)imide (LiTFSI) in water and the ionic liquid 1-ethyl-3-methylimidazolium bis(trifluoromethylsulfonyl)imide (EMIM-TFSI). Finally, the exceptionally strong wetting response of a biphasic system (electrolytes surrounded by an immiscible liquid phase) is described. The general experimental configuration is illustrated schematically in Figure 1b,c for the single and biphasic configurations, respectively. In each case, the effect of the underlying electrochemical processes on the apparent contact angle, θ, at the HOPG–electrolyte interface in air is investigated, and we propose strategies to implement the acquired knowledge toward the development of biphasic systems for use in electrowetting-based optofluidic devices. The aim of the work is to provide fundamental insights into the understanding of carbon–electrolyte interactions under external potential bias, while highlighting the promising character of carbon-based materials for applications in various types of electrowetting-driven microfluidic devices.

## Results and Discussion

### Liquid–Air Electrowetting Using Organic Electrolytes

**Figure 2:**
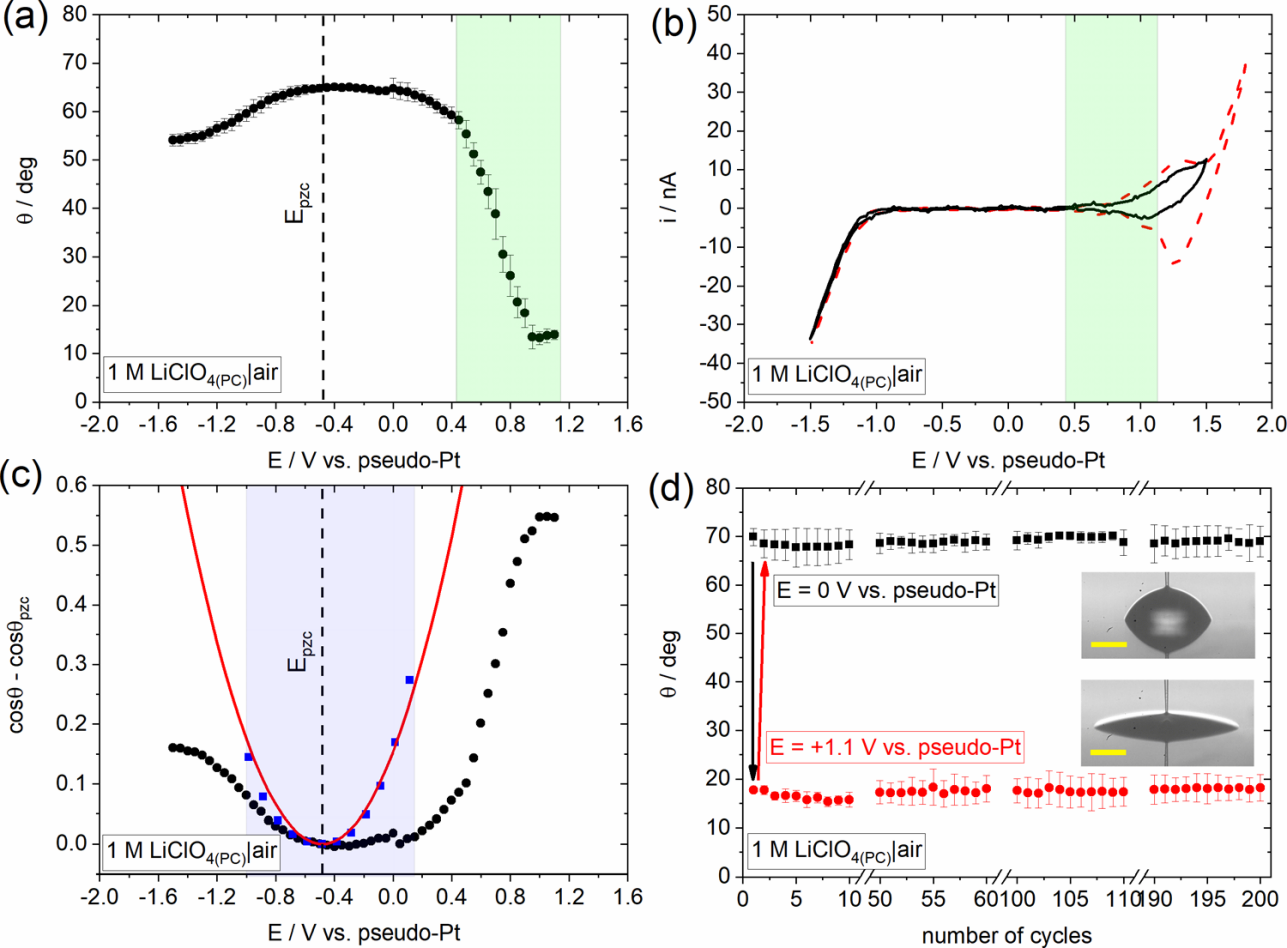
(a) Change in apparent equilibrium electrowetting contact angle, θ, with the applied bias (values are reported vs Pt wire pseudo-reference electrode) at the HOPG–1 M LiClO_4(PC)_ interface in air. Measurements were conducted under static conditions based on the protocol described in Experimental Section 1.3 in the Supporting Information. (b) Cyclic voltammogram recorded in a sessile droplet of 1 M LiClO_4(PC)_ deposited on HOPG, at 100 mV s^–1^. In panels (a) and (b), the highlighted regions are used to indicate the matching potential window between θ vs *Ε* plot and CV curves. (c) Electrowetting curves based on the experimental data presented in panel (a) (black dots). Also shown is the cos θ – cos θ_eq_ difference obtained by applying the Young–Lippmann equation to the experimental capacitance data for the 1 M LiClO_4(PC)_ solution and the interfacial surface tension for the 1 M LiClO_4(PC)_–air interface (see Figure 3 and Table S1, blue squares). The theoretical response in the whole potential window studied (red lines) was approximated by fitting a quadratic function of the potential to the experimentally obtained response (see Section S2.1). The highlighted region corresponds to the purely capacitive potential window as was determined by EIS measurements. (d) Changes in apparent electrowetting contact angle, θ, during wetting/dewetting cycles at the HOPG–1 M LiClO_4(PC)_ interface in air following the protocol described in the Experimental Section in the Supporting Information. One cycle corresponds to two consecutive potential pulses from 0 to +1.1 V vs pseudo-Pt. The changes in θ were extracted by recording the dynamics of the advancing/receding motions using a frame rate of 50 fps (see Movie S1). Inset: Droplet images at applied biases of 0 and +1.1 V vs pseudo-Pt. Scalebars correspond to 100 μm.

**Figure 3:**
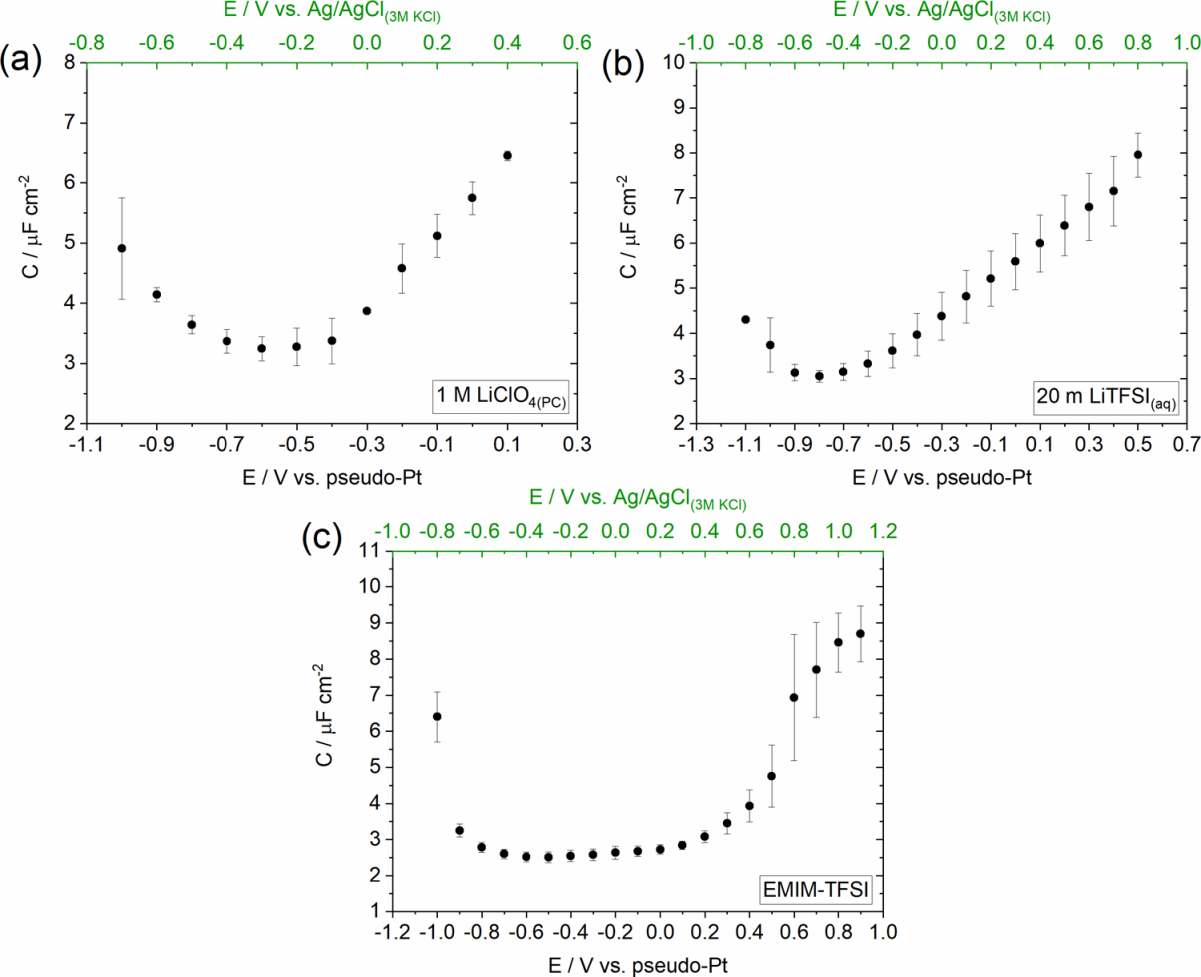
Dependence of HOPG capacitance on the applied potential for (a) 1 M LiClO_4(PC)_, (b) 20 m LiTFSI_(aq)_, and (c) EMIM-TFSI electrolytes. Capacitance values were extracted from electrochemical impedance spectroscopy (EIS) measurements adopting the approach described in the Experimental Section in the Supporting Information. EIS spectra were recorded in the frequency range between 20 kHz and 1 Hz with an imposed AC rms (root-mean-square) amplitude of 7 mV peak-to-peak. The depicted data shows averages of three to five experiments. A capacitive response was considered for phase angle values higher than 85°. The experiments were performed using the Teflon cell setup (see Figure 1c) on freshly cleaved HOPG samples. The potential scale was converted from the Ag/AgCl_(3 M KCl)_ reference electrode to the pseudo-Pt reference based on the procedure described in the Experimental Section in the Supporting Information.

The contact angle of 1 M LiClO_4_ in PC (denoted hereafter as LiClO_4(PC)_) organic electrolyte droplet on HOPG was monitored as a function of the potential, *E*, applied vs a Pt wire pseudo-reference electrode using the setup displayed in Figure 1a and following the experimental protocol described in the Experimental Section in the Supporting Information. Figure 2a shows the electrowetting responses of the equilibrium contact angle within a potential window of 2.6 V. On first inspection, significant variations in contact angle with *E* are observed, up to a maximum change of ca. 50° vs its equilibrium value, θ_pzc_, at the potential of zero charge, *E*_pzc_, located at ca. −0.48 V. The latter has been estimated by the minimum in the capacitance plot presented in Figure 3a. Similar contact angle changes have been previously reported for a series of relatively concentrated (3 M) alkali chlorides aqueous electrolytes on HOPG,^43^ with however, a threshold voltage ca. twice that recorded in the present work. This finding indicates that the electrowetting response is amplified at the HOPG–LiClO_4(PC)_ interface possibly due to the effect of an underlying potential-dependent surface process. An additional, notable characteristic of the electrowetting curves is the strong asymmetry relative to *E*_pzc_. In particular, the recorded contact angle for *E* < *E*_pzc_ reaches a maximum change of ca. 10° relative to θ_pzc_ at −1.3 V, with subsequent contact angle saturation at more negative potential biases. This implies that, in contrast to the observations at *E* > *E*_pzc_, parallel processes, which are most likely to be faradaic reactions, suppress the (capacitively driven) electrowetting response of the electrolyte.

To investigate the possibility of charge transfer reactions occurring on the surface of graphite, the current in the applied potential window was monitored by cyclic voltammetry (CV); see the results presented in Figure 2b. For the anodic scan up to the potential limit of +1.5 V, an oxidation wave is detected from ca. +0.3 to +0.4 V, with an ill-defined shoulder located at ca. +1.3 V and a reduction peak at ca. +1.02 V during the reverse scan. Upon further positive polarization up to +1.8 V, the oxidation peak at +1.3 V becomes more pronounced and a distinct reduction counterpart is recorded at ca. +1.27 V in the reverse potential sweep. The development of the reduction peak with an increase in the positive potential limit clearly indicates the correlation between the two processes detected for *E*> 0.2 V. Therefore, the possibility of a CO_2_ evolution reaction (or other carbonaceous byproducts such as propanal, propylene oxide, acetone, and 2-ethyl-4-methyl-dioxolane) arising from the decomposition of PC^47,48^ and/or oxygen evolution reaction (OER) due to the presence of water impurities in the solvent^49^ is rather unlikely since a reduction counterpart is not expected for these processes. Furthermore, in the electrolyte medium used, such reactions require the application of significantly higher overpotentials.^48,50^ In this respect, we attribute the observed overall process to the intercalation/deintercalation of ClO_4_^–^ anions into graphite, which is consistent with previous reports for aqueous^51−54^ and non-aqueous^55−57^ electrolytes (refer also to the next section). The process is driven through the exposed step edges on HOPG^53^ and can be considered as the main cause for the enhanced electrowetting response seen within this potential region. Similar to what is reported for aqueous electrolytes,^52,54^ we ascribe the peaks recorded when the potential limit was restricted to +1.5 V (black solid line in Figure 2b) to early stages of intercalation, i.e., dilute intercalation (involving an initial adsorption step), with the onset of more advanced stages upon further positive polarization (red dashed line in Figure 2b). Zhang et al.^58^ have also reported the effect of ClO_4_^–^ anion intercalation into HOPG on the electrowetting response of 1 mM and 0.1 M NaClO_4_ in water, with, however, significantly smaller contact angle changes compared to what is reported in our study. This can be ascribed to the simultaneous occurrence of OER in aqueous solutions that is expected to have a negative effect on the overall process. Another possible explanation for the enhancement of the electrowetting response in the non-aqueous system, is the weak solvation of ClO_4_^–^ ions in PC compared to water,^59^ which results in an appropriate difference between intercalation and hydration energies^60^ and therefore facilitates the intercalation process inside the interlayer galleries of graphite, similar to what is reported for anions such as BF_4_^–^.^61^ Furthermore, the lower donor number of PC (63.1 kJ mol^–1^)^62^ compared to water (75 kJ mol^–1^),^63^ may reduce slightly the negative surface charge of any edge sites,^64^ and consequently weaken the repulsive Coulombic forces between the latter and the ClO_4_^–^ ions,^58^ a phenomenon that is expected to further promote the intercalation process. The intercalation of ClO_4_^–^ ions into graphite, is also anticipated to increase the in-plane conductivity of the outermost graphene layers similar to processes reported in the literature for various graphite intercalation compounds (GICs).^65,66^ Since electrowetting is strictly a surface phenomenon, even dilute intercalation should be sufficient to decrease the ohmic drop at the graphite–electrolyte interface. Changes in the potential of the interface are expected to influence the electrowetting response, as based on the Young–Lippmann (Y–L) equation (eq 1; where *C* and γ_LV_ are the capacitance of the interface and the liquid–air interfacial tension respectively), the difference between the cosine of the CA at an applied bias not equal to *E*_pzc_, cos θ, and that at *E*_pzc_, cos θ_pzc_, exhibits a quadratic dependence on the applied potential, *E*.^16^
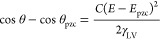
1

During the cathodic potential sweep, a relatively wide capacitive potential window is recorded ranging from ca. +0.2 to −1.0 V, followed by a sharp increase in current (in absolute value) at more negative potential biases. The latter is ascribed to the cathodic decomposition of the solvent with a possible contribution from the hydrogen evolution reaction (HER) triggered by water impurities (since the experiments were conducted at ambient temperature).^47,67^ In more detail, the overall cathodic process involves the evolution of CO_2_ due to the water catalyzed ring-opening of PC as well as the formation of other byproducts such as carbonates.^67^ Returning to the θ vs *E* plots in Figure 2a, it is evident that the increase in the rate of the above mentioned processes with *E* inhibits the electrowetting response, a phenomenon depicted in the low CA changes recorded for *E* < *E*_pzc_ and is further highlighted in the contact angle saturation observed for *E* < −1.2 V. Therefore, we can conclude that solvent decomposition occurring at *E* < −0.8 V is detrimental to the electrowetting phenomenon due to the electrochemical modification of the surface of HOPG as a result of the byproducts (often insoluble) formed upon solvent decomposition^47,67^ that increase the solid–electrolyte interfacial surface tension.^68^

To further confirm that the underlying electrochemically induced processes occurring at the surface of HOPG drive the mechanism of electrowetting, we examine the agreement of the experimentally recorded contact angle changes (displayed in Figure 2a) with those predicted by the Y–L equation (eq 1). Figure 2c shows a direct comparison between the electrowetting curve of the experimental data presented in Figure 2a (black dots) and that estimated by the Y–L equation using the experimentally determined *C* and γ_LV_ values (blue squares; see Section S2.2). The theoretical Δcos θ values in the potential region under study (red line) were estimated by fitting a quadratic function of *E* to the electrowetting response calculated by eq 1 (blue squares), using an average effective capacitance as an approximation. It can be seen that the Y–L equation captures the experimental response only within a narrow potential range (less than 500 mV) close to *E*_pzc_. At potential biases outside this region, the experimental response strongly deviates from the predictions of the Y–L equation. This finding demonstrates that the mechanism of electrowetting in the case of a conductive substrate is very sensitive to the underlying electrochemically induced processes and therefore is different to the case of EWOD, where the CA changes exhibit a strong dependence on the capacitance of the interface (being dependent on the thickness of the dielectric layer), with a straightforward relation to the applied potential following the Y–L equation. In other words, these apparent discrepancies between the experimental data and the predictions of the Y–L equation can be explained by the breakdown of the polarizable character of the interface (being a fundamental precondition for the application of the Y–L equation).^35^ The occurrence of faradaic (redox) reactions, i.e., charge transfer due to adsorption/intercalation of anions on/into graphite strongly alters the physicochemical properties of the substrate, and the interface exhibits a non-ideally polarizable character. These phenomena are not considered in the derivation of the Y–L equation and hence the latter fails to describe the obtained experimental response.

Following the study of the interfacial processes at the HOPG–LiClO_4(PC)_ interface and their effect on the electrowetting response, we proceed by examining the reversibility of the phenomenon. Figure 2d shows the contact angle variations among 200 consecutive wetting/dewetting cycles between 0 and +1.1 V (see Experimental Section in the Supporting Information). The positive potential limit was chosen so that it lies within the potential region where intercalation occurs and the maximum contact angle changes are observed (Figure 1a). Overall, highly reproducible changes (to within less than 1.5% over 200 cycles) of contact angle are obtained, which demonstrates the highly reversible character of the ClO_4_^–^ intercalation-driven electrowetting process (see Movie S1).

### Liquid–Air Electrowetting Using Concentrated Aqueous Electrolytes and Ionic Liquids

**Figure 4:**
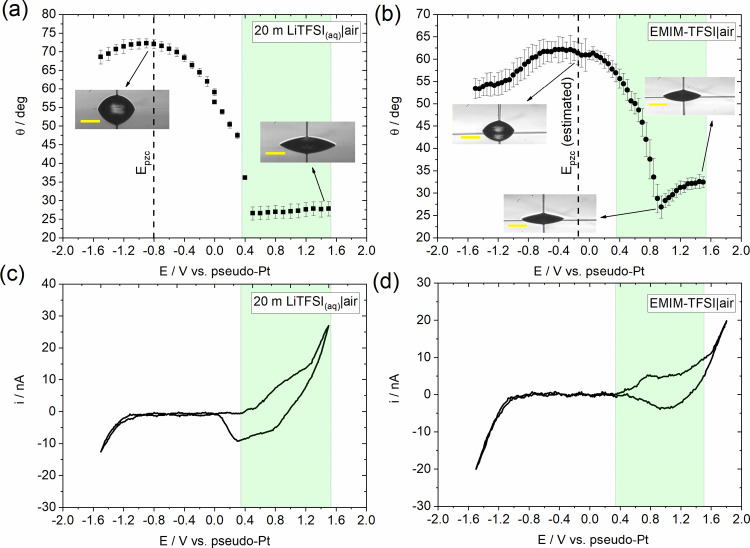
(a, b) Change in apparent equilibrium electrowetting contact angle, θ, with the applied bias (values are reported vs Pt wire pseudo-reference electrode) at (a) HOPG–20 m LiTFSI_(aq)_ and (b) HOPG–EMIM-TFSI interfaces in air. Measurements were conducted under static conditions based on the protocol described in the Experimental Section in the Supporting Information. Insets: Droplet images at selected applied biases indicated by the arrows. Scalebars correspond to 100 μm. Cyclic voltammogram recorded in a sessile droplet of (c) 20 m LiTFSI_(aq)_ and (d) EMIM-TFSI deposited on HOPG using a scan rate of 100 mV s^–1^. The highlighted regions are used to indicate the matching potential window between θ vs *Ε* plots and CV curves.

Having characterized the electrowetting process in the presence of intercalating anions from an organic electrolyte, we proceed to investigate the effect of different solvents on the phenomenon. Toward that end, we use the highly concentrated aqueous electrolyte 20 m LiTFSI (denoted hereafter as 20 m LiTFSI_(aq)_, with concentrated compositions denoted in molal terms) and the ionic liquid EMIM-TFSI. We focus on concentrated electrolytes in order to expand the potential window and promote anion intercalation into graphite.^60^Figure 4a,b shows the contact angle variations with *E* of the 20 m LiTFSI_(aq)_ and EMIM-TFSI sessile drops, respectively, deposited on HOPG in air. For both electrolytes, at applied potential biases more positive than *E*_pzc_ (the latter is located at ca. −0.8 V for the 20 m LiTFSI_(aq)_ and at ca. −0.11 V for the EMIM-TFSI; see Figure 3 and Section S2.1), significant contact angle changes are observed within less than +1.5 V vs *E*_pzc_. In more detail, for the aqueous electrolyte, a maximum contact angle change of ca. 45° vs θ_pzc_ is recorded at +0.5 V, while further positive polarization results in contact angle saturation. In the case of the ionic liquid, the maximum contact angle change is seen at ca. +0.95 V, followed by a particularly noteworthy increase in contact angle by ca. 5° at *E* > +0.95 V with subsequent contact angle saturation (discussed in more detail below). Upon application of negative potential biases with respect to *E*_pzc_, relatively minor variations in contact angle (less than 10°) are seen. Overall, these findings are reminiscent of the electrowetting response recorded at the HOPG–1 M LiClO_4(PC)_ interface (Figure 2) and therefore imply a strong effect of the electrolyte–graphite interactions induced by the applied potential bias on electrowetting.

Following a similar approach to the aforementioned analysis of the LiClO_4(PC)_ organic electrolyte, the electrochemical processes occurring on the surface of HOPG for the aqueous electrolyte and the ionic liquid were initially investigated using CV measurements. Figure 4c,d presents the cyclic voltammograms recorded in a droplet of 20 m LiTFSI_(aq)_ and EMIM-TFSI, respectively, deposited on the surface of HOPG. During the anodic potential sweep, a distinct oxidation process is detected at ca. +0.4 V for both electrolytes, with a continuously increased rate up to the positive applied potential limit (i.e., +1.5 V for the 20 m LiTFSI_(aq)_ and +1.8 V for the EMIM-TFSI). Reversing the potential scan direction gives rise to a merged reduction with peaks at ca. +0.75 and +0.3 V for the aqueous electrolyte and a well-defined cathodic peak at +1 V for the ionic liquid. These quasi-reversible processes are attributed to TFSI^–^ adsorption and intercalation/deintercalation into graphite, in line with what is reported in the literature for highly concentrated aqueous electrolytes^60^ and ionic liquids^69−71^ using graphite electrodes. As described previously for the intercalation of ClO_4_^–^ from 1 M LiClO_4(PC)_, the process is expected to be initiated through the exposed edge sites on the surface of HOPG and is believed to drive the electrowetting response for *E* > *E*_pzc_. The possibility of electrolyte decomposition upon positive polarization in the aqueous solution is expected to be minimal, since within the applied potential window, OER is unlikely to occur due to the low water-to-salt molar ratio in the highly concentrated 20 m LiTFSI_(aq)_ electrolyte.^60^ Similarly, OER arising due to water impurities in the ionic liquid requires overpotentials that exceed the anodic potential limit used and hence its contribution can be disregarded.^72^ The cathodic reactions identified at *E* < *E*_pzc_ in the cyclic voltammograms shown in Figure 4c,d at ca. −1 V for both electrolytes can be assigned to HER with a minor (if any) contribution from the reductive decomposition of TFSI^–^ for the aqueous electrolyte and the cathodic decomposition of the ionic liquid with contributions from HER for the EMIM-TFSI electrolyte. In a similar way to what is observed for LiClO_4(PC)_ (see the discussion in the relevant section), these processes appear to suppress electrowetting.

**Figure 5:**
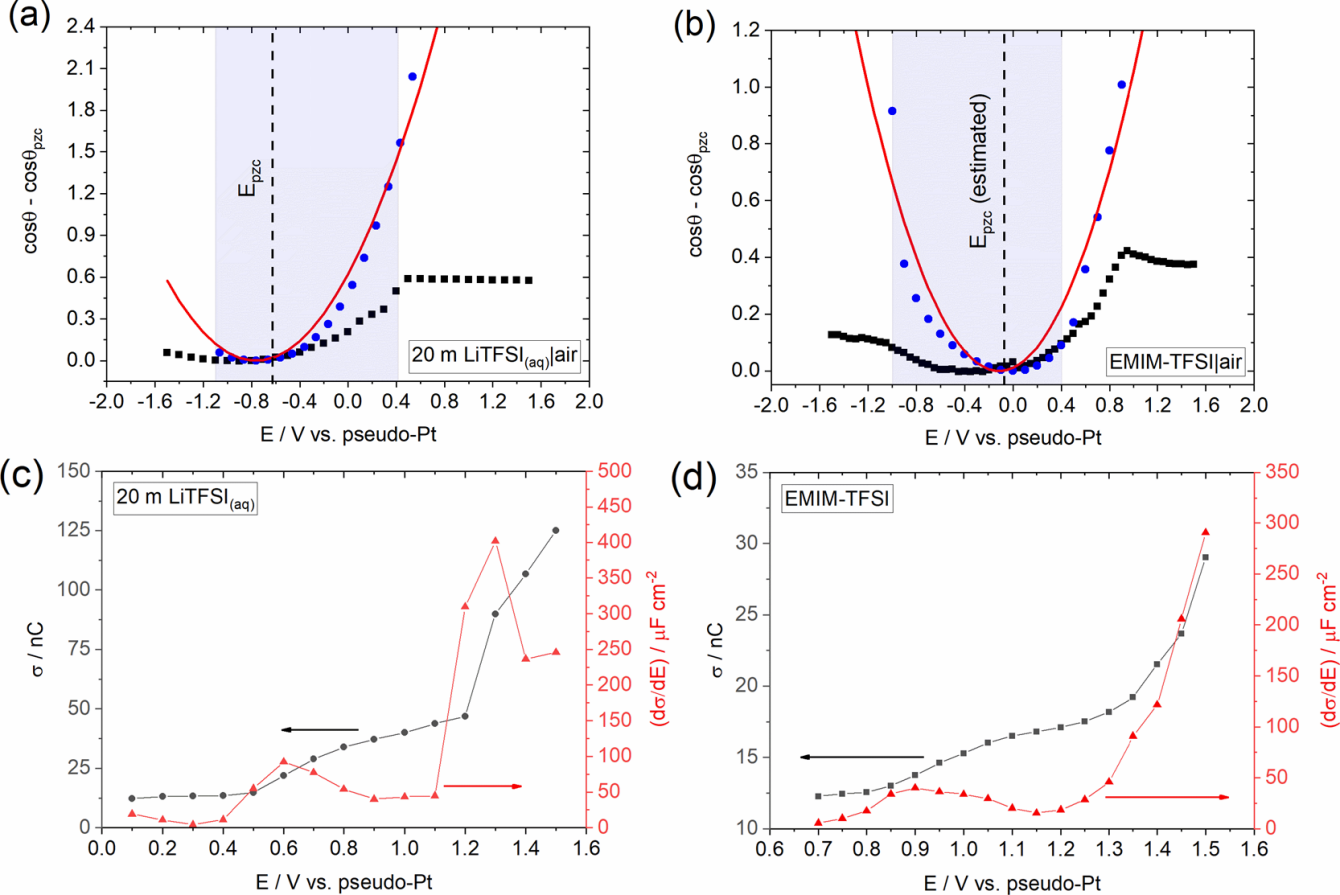
Electrowetting curves based on the experimental data presented in Figure 4a,b (black squares) for (a) 20 m LiTFSI_(aq)_ and (b) EMIM-TFSI electrolytes. In the same figures, the cos θ – cos θ_eq_ difference obtained by applying the Young–Lippmann equation using the capacitance data determined experimentally within the purely capacitive window for the two electrolytes and the corresponding interfacial surface tensions (see Figure 3 and Table S1) is also given (blue circles). The theoretical response in the whole potential window studied (red lines) was approximated by fitting a quadratic function of the potential to the experimentally obtained response (see Section S2.1). The highlighted regions correspond to the purely capacitive potential window as that was determined by EIS measurements. Variations in surface charge, σ, with the applied potential bias, *E*, at the (c) 20 m LiTFSI_(aq)_–HOPG and (d) EMIM-TFSI–HOPG interfaces. The charge was extracted by integration of the current vs time curves recorded at each individual potential pulse during the static electrowetting experiments. In the same plots, the variation of the parameter dσ/d*E* with *E* is also given. Since the former represents the capacitance of the interface, the data were normalized per electrode nominal area. This was calculated by image analysis of each individual droplet at the corresponding *E*, based on its equilibrium diameter (extracted using the custom-made algorithm and considering a circular contact area). For further details on the potential pulse sequence and image analysis, see the Experimental Section of the Supporting Information.

In Figure 5, we compare the electrowetting curves (black squares) obtained from the experimental response of the two electrolytes (presented in Figure 4a,b) with the theoretical predictions of the Y–L equation (eq 1) using the experimentally determined *C* and γ_LV_ values as described in the first section (blue circles; see Section S2.1). The data demonstrate the strong divergence of the experimental response from the Y–L equation within most of the applied potential window, with the exception of a narrow range in the vicinity of *E*_pzc_, i.e., less than 600 mV. As in the case of LiClO_4(PC)_, this finding further highlights the fact that the mechanism of electrowetting on HOPG in the presence of electroactive species (such as intercalating ions) is governed by the potential-dependent charge transfer processes and not just the changes in *C* with *E* and the variations of the latter (EWOD systems). Depending on the effect of these processes on the physicochemical properties of the substrate near its surface and hence on the energetics at the interface (i.e., the potential-dependent solid–electrolyte interfacial surface tension), electrowetting can be either enhanced (e.g., anion intercalation) or suppressed (e.g., electrolyte decomposition).

Revisiting the electrowetting response of the ionic liquid electrolyte presented in Figure 4b, we focus on providing insights into the origin of the minimum in the contact angle located at +0.95 V in the θ vs *E* plot. A sensible starting point is to treat the data phenomenologically and attempt to identify possible underlying effects during the charging of the interface on the potential dependent solid–electrolyte interfacial surface tension (and through that to *C*), similar to the early approaches in electrocapillarity studies. On this basis, since such effects are expected to influence the surface charge, σ, we monitor the variations of the latter with *E* within the potential window where the minimum in contact angle is observed. The data were derived by integration of the current vs time curves recorded for each individual potential pulse applied during the static electrowetting measurements of Figure 4b (see Experimental Section in the Supporting Information). From the results displayed in Figure 5d, it can be seen that the rate of charge storage at the interface varies significantly with *E*. To quantify these variations, we numerically differentiated the experimental σ values with respect to *E* and the results are plotted in the same figure. The parameter dσ/d*E* represents the capacitance of the interface, and hence it may be used to provide information about the processes associated with the reconstruction of the electrode’s surface. Note that the values are reported per electrode nominal surface area, as calculated by image analysis of each individual droplet at the corresponding *E*, based on its equilibrium diameter (extracted using the custom-made algorithm described in the Experimental Section) and considering a circular contact area. The most interesting feature in the dσ/d*E* vs *E* plot is the hump observed at ca. +0.9 V, which almost coincides with the minimum in the θ vs *E* curves of Figure 4b. We attribute the decrease in dσ/d*E* parameter (and thus *C*) observed in the +0.95 to +1.2 V potential range to the electrostatic interplay between the different conformers of the adsorbed ionic liquid and the electrode, in line with what is reported in the literature.^73,74^ Upon increasing the potential bias above +1.2 V, the strongly adsorbed (as evidenced by the decrease in *C*) TFSI^–^ anions and EMIM^+^TFSI^–^ ion pairs start to intercalate into graphite (the latter dissociate fully due to the increased electrostatic attractions with graphite), which results in the significantly enhanced capacitance at the interface. At the same time, more active sites are generated at the surface of the electrode and the initial adsorption step is facilitated. A similar behavior is observed for the concentrated aqueous electrolyte (Figure 5c), with the hump at ca. +0.6 V in the dσ/d*E* vs *E* plot concurring with the starting point of contact angle saturation in Figure 4a. In line with what is described above, we ascribe this feature to the electrostatic interactions between the strongly adsorbed TFSI^–^ anions on the surface of HOPG. Another notable feature in the same plot is the second hump seen at higher potentials, i.e., +1.3 V. Considering that TFSI^–^ anion intercalation from aqueous solutions cannot proceed easily to the more advanced stages of intercalation due to the potential window of the aqueous electrolyte,^60^ this finding might indicate the maximum stage of intercalation for the system. Exceeding this point, as more TFSI^–^ anions adsorb on the surface of the electrode, the electrostatic interactions among the adsorbates increase (since the rate of intercalation drops and the ions accumulate on the surface of the electrode) and the system approaches “surface charge saturation.” The latter might also explain the contact angle saturation observed in Figure 4a.

An additional important parameter is the effect of potential-induced structural changes on the electrowetting response of graphite. It is well-established (both theoretically and experimentally) that intercalation processes involve phase transition from the initial dilute stages of intercalation (e.g., stage 4) to more advanced stages (e.g., stages 3 and 2).^75^ As intercalation proceeds with more ions filling the interlayer space in between the graphene sheets, lattice expansion occurs which, depending on the size of the intercalant, results in structural changes of varied extent.^60,75^ For example, it is reported that stage 2 to 1 TFSI^–^-GICs formed in the ionic liquid electrolyte 1-Butyl-1-methylpyrrolidinium bis(trifluoromethylsulfonyl) imide (Pyr_14_TFSI) exhibit a gallery expansion of ca. 137%,^75^ while stage 4 to 2 TFSI^–^-GICs formed in aqueous electrolytes show values in between ca. 137–143%.^60^ In contrast, intercalation of the smaller ClO_4_^–^ anion into graphite from PC leads to a lattice expansion of ca. 90%.^55^ Such extensive structural modifications generated during the intercalation process are expected to influence the electrowetting response by introducing surface heterogeneity, the extent of which increases with staging of intercalation. Under these conditions and in parallel to the charging phenomena previously analyzed, a transition to the Wenzel wetting state^76^ might occur.

**Figure 6:**
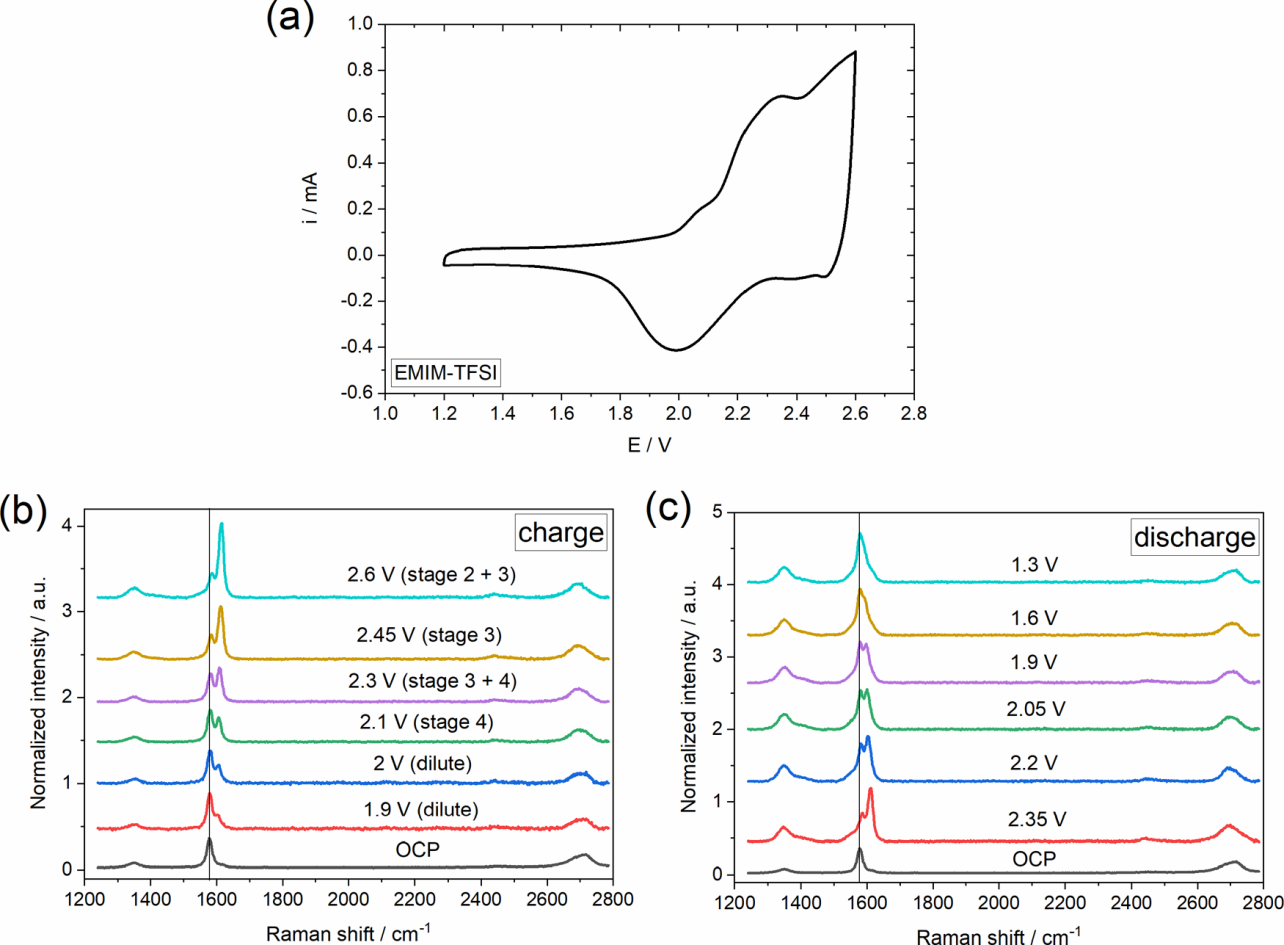
(a) Cyclic voltammogram recorded at a scan rate of 10 mV s^–1^ in EMIM-TFSI using a coin-type cell (see the Experimental Section in the Supporting Information) prepared from a KS4 graphite positive electrode and a commercial activated carbon negative electrode. The voltage was scanned between 1.2 V (start potential) and 2.6 V. In situ Raman spectral series for the TFSI^–^ insertion during the (b) charge and (c) discharge reactions. The potential corresponding to each individual spectrum is labeled accordingly. The stage index of intercalation is also indicated as that was estimated by the ratio of the normalized intensities *I*_i_/*I*_b_, where *I*_i_ and *I*_b_ correspond to the intensities of the *E*_2g2i_ and *E*_2g2b_ bands respectively.^77^

Raman spectroscopy has been widely used to probe the charge transfer processes during the insertion of various intercalants into graphitic materials.^77−79^ To this end, we evaluate the above mentioned hypothesis by systematically studying the structural changes upon insertion of the TFSI^–^ anion into graphite, via in situ Raman measurements during the charge and discharge of a graphite based coin-type cell. The latter was prepared using KS4 graphite (as a model system of sp^2^-hybridized carbon; the sp^2^ hybridization is evidenced in the low intensity of the D peak located at ca. 1348 cm^–1^ in the Raman spectrum recorded at open circuit potential (OCP), see Figure 6b) for the positive electrode and activated carbon (AC) for the negative electrode (see Experimental Section in the Supporting Information). The cyclic voltammogram recorded at the KS4 cathode in EMIM-TFSI using the above mentioned coin cell configuration is presented in Figure 6a. As the applied potential is scanned in the positive direction, an oxidation wave at ca. +1.75 V is recorded, followed by three shoulder peaks in the potential range of +2 to +2.6 V due to staged intercalation of TFSI^–^.^71^ During the reverse scan direction, one well-defined reduction peak at ca. +1.98 V and a set of unresolved peaks at more positive potentials are seen that are attributed to the extraction of TFSI^–^. Figure 6b,c shows the in situ Raman spectra obtained for the freestanding KS4 cathode in EMIM-TFSI during the charge and discharge cycles of the cyclic voltammogram presented in Figure 6a. At OCP, the characteristic G band appears at 1579 cm^–1^ associated with the *E*_2g2_ symmetry due to the in-plane vibration of sp^2^ carbon atoms in graphite,^80^ accompanied by the 2D band at ca. 2704 cm^–1^. In the same spectrum, a weak D band is also observed at ca. 1348 cm^–1^, indicating that the electrode preparation did not introduce any significant defects on the graphite film. Any charge transfer occurring during the formation of GICs that induces lattice expansion will strongly influence the G band. In particular, changes in the interlayer space of the graphene sheets taking place throughout the successive stages of intercalation will lead to G band splitting. The interior unintercalated layers will exhibit the original *E*_2g2i_ mode at ca. 1580 cm^–1^, while the bounding layers close to the intercalated ions will respond at an upshifted *E*_2g2b_ mode,^65,79^ the exact extent of which is determined by the stage index of intercalation. When the cell is charged at +1.9 V, the G band splits into two Raman modes, *E*_2g2i_ and *E*_2g2b_. Upon increasing charging potential, the intensity of the *E*_2g2i_ band decreases and the *E*_2g2b_ band becomes more evident due to the evolution of ordered staging. At applied potentials more positive than the second oxidation wave in the cyclic voltammogram of Figure 6a, the *E*_2g2b_ band overtakes the intensity of *E*_2g2i_. A blue shift in *E*_2g2b_ band from ca. 1602 cm^–1^ at +1.9 V to 1617 cm^–1^ at +2.6 V is also observed, which indicates the transition to more advanced intercalation staging. At the maximum charging potential, i.e., +2.6 V, corresponding to the third oxidation peak in Figure 6a, the ratio of the normalized intensities *E*_2g2i_ to *E*_2g2b_, *I*_i_/*I*_b_, equals ca. 0.33. From this ratio, the stage index of intercalation is calculated to be ca. 2.65, indicating the formation of stage 2 + 3 GICs (assuming the ratio of the cross sections for Raman scattering of the inner and boundary layers is unity).^77^ Finally, the position of the 2D band at +2.6 V is red-shifted by ca. 12 cm^–1^, which is consistent with the transition to more advanced stages of intercalation.^79^

During discharge, the deintercalation of TFSI^–^ anions leads to the reverse trend. A decrease in the applied potential results in a gradual decrease in the intensity of the *E*_2g2b_ band accompanied by a continuously re-emerging *E*_2g2i_ mode. After full discharge of the cell (i.e., at +1.3 V), the *E*_2g2i_ mode re-appears completely, indicating the successful extraction of the TFSI^–^ anions. However, the ill-defined shoulder in the immediate vicinity of the *E*_2g2i_ mode that appeared after charging/discharging of the cell and the increase in the intensity of the D band by a factor of ca. 4 relative to its value at OCP (prior to charging/discharging of the cell) show that the overall process of intercalation/deintercalation introduces defects in the original graphitic structure.

Having investigated the physicochemical processes occurring at the interface between graphite and EMIM-TFSI by both electrochemical means (Figure 5) and by in situ Raman spectroscopy (Figure 6), we proceed to identify the combined effect of electrostatic interactions and structural changes as the main factor being responsible for the minimum observed in Figure 4b. This is reasonable, since by considering the physical connection between electrocapillarity measurements and electrowetting, changes in the capacitance of the interface due to interfacial processes, such as adsorption, are expected to change the sign of the dependence of θ on *E*. Considering the thermodynamic definition of double layer capacitance as the negative second partial derivative of the electrode–electrolyte interfacial surface tension,^81^ the presence of an inflection point in the electrowetting curves implies that capacitance takes negative values. However, such charge dissipation phenomena (arising by, e.g., conductivity variations^82^ or electrode poisoning by inactive adsorbates^83^) have not been identified in our measurements (the dσ/d*E* does not exhibit negative values in Figure 5c,d).

The structural changes identified during the intercalation/deintercalation of TFSI^–^ into graphite as well as the effect of the surface charge variations with potential described earlier (see Figure 5 and the discussion therein) demonstrate that electrowetting acts as a macroscopic probe of such surface processes in sp^2^-hybridized graphite. A depiction of this phenomenon is presented in Movie S2. The video shows the response of an EMIM-TFSI droplet on HOPG in air, after stepping the potential from +1.7 V (held for 200 s) to 0 V. Based on the cyclic voltammogram shown in Figure 4d, the former value corresponds to the potential region within which staged intercalation occurs. Remarkably, it can be seen that upon discharging of the interface, the droplet vibrates on the surface of the electrode until it reaches an equilibrium. The “dance” of the droplet is the result of the reconstruction of the surface due to the deintercalation of TFSI^–^ anions, in line with what has been proved earlier by Raman spectroscopy (see Figure 6 and the discussion vide supra). In parallel, the surface charge heterogeneity introduced due to the ongoing structural changes during deintercalation also contributes to this phenomenon. Such variations in surface charge will result in the generation of an inhomogeneous potential distribution on the surface of graphite, which is expected to influence the electrowetting response by introducing variations in the surface energy, being reflected in the observed contact angle perturbations. In other words, in complete analogy with electrowetting-based lab-on-a-chip devices where changes in the applied voltage induce droplet motion, the variations of surface potential drive a vibrational movement of the droplet. The final equilibrium value of the contact angle at 0 V is determined to be almost identical to that before the positive potential pulse (to within less than 0.7%; see Figure S3). The phenomenon was reproducible over three consecutive cycles (see Figure S3), demonstrating its reversibility and highlighting the sensitivity of electrowetting to the structural changes induced by anion intercalation. The same response was also recorded for the concentrated aqueous electrolyte of 20 m LiTFSI_(aq)_ (see Movie S3), but not for the 1 M LiClO_4(PC)_. This can be explained by the smaller size of ClO_4_^–^ anion, which results in a lower extent of structural changes during the intercalation process.^52,55^

Summarizing the results of the first two sections, we can conclude that anion intercalation into HOPG occurring based on the general reaction scheme presented below (eq 2) drives the electrowetting process at *E* > *E*_pzc_.
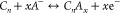
2

Depending on the stage of intercalation and the size of anions, the original graphitic structure can be fully or partially recovered. In the case of the smaller ClO_4_^–^ anion and at applied biases corresponding to the early stages of intercalation, i.e., dilute intercalation, electrowetting is fully reversible and fast (see Figure 2d; refer also to the next section as well as Figure S5 and the relevant discussion in the Supporting Information). On the other hand, the larger TFSI^–^ anion causes larger lattice expansion in the galleries of graphite, and during advanced intercalation stages, i.e., stage 3 and 2 + 3, defects are also introduced (see Figure 6). Furthermore, strong electrostatic interactions between the TFSI^–^ anions are observed, which influence the surface charge. Consequently, electrowetting exhibits “irregular” variations in the contact angle with subsequent contact angle saturation within the potential region, where these processes occur (see Figure 4), while the overall process is slower (yet reversible in short term) due to the reconstruction of the surface (see the discussion above). At this point, it is worth highlighting the fact that the enhancement of electrowetting due to the intercalation of anions into HOPG demonstrates that, in strong contrast to what is well-established in EWOD technology, charge transfer processes (or charge injection using EWOD terminology) can have a beneficial effect in the case of electrowetting directly on conductors when certain conditions are satisfied. These conditions require that the physicochemical changes induced by the application of potential bias change the energetics of the system in a reversible way that also promotes electrowetting.^35,45^ In our case, anion intercalation alters the energetics of the interface by largely lowering the interfacial surface tension, while simultaneously increasing the in-plane conductivity of the outermost graphene layers on HOPG. Both phenomena are responsible for the observed significant enhancement of electrowetting at *E* > *E*_pzc_.

### Biphasic Systems for Liquid–Liquid Electrowetting

**Figure 7:**
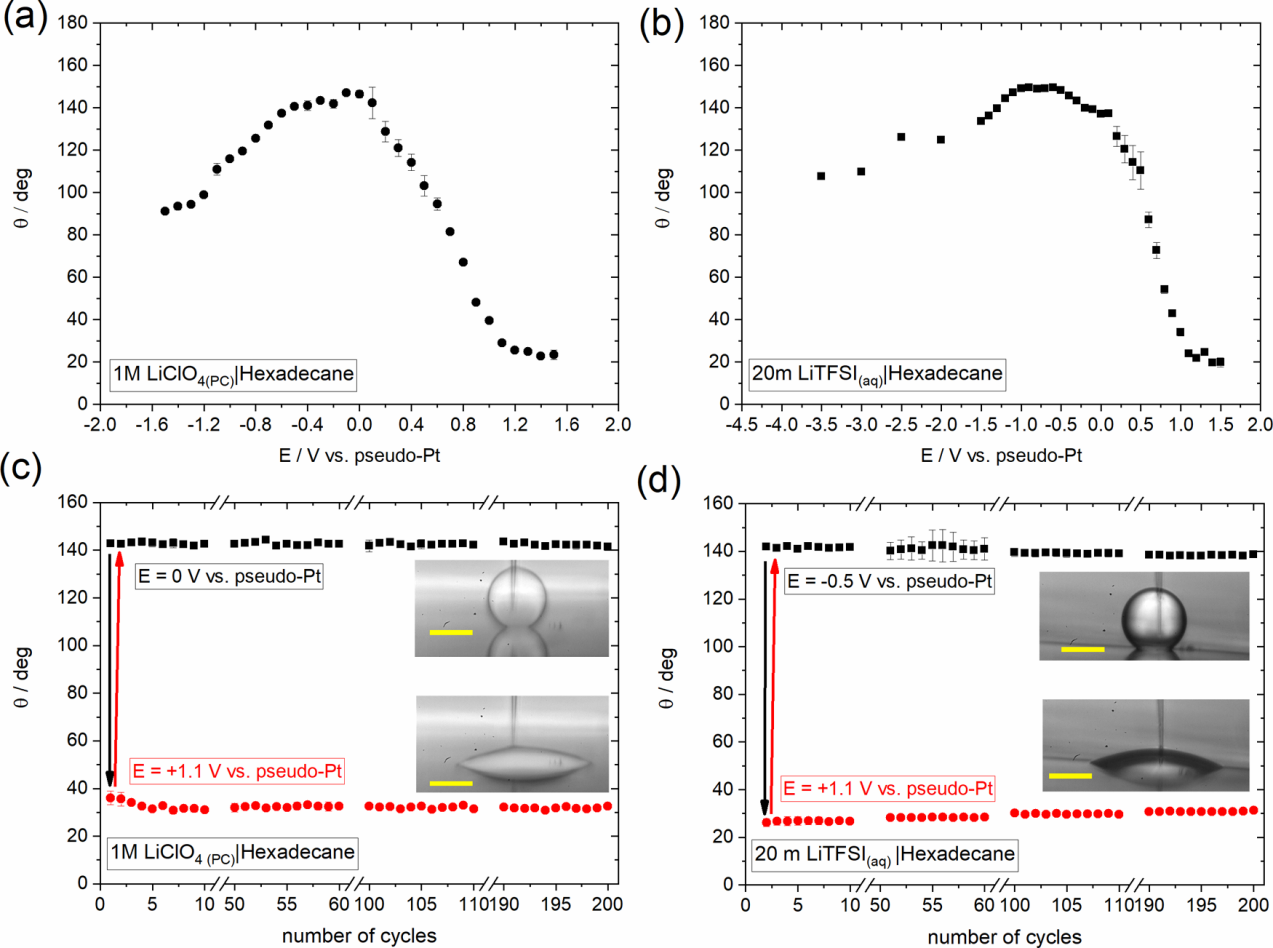
Change in the apparent equilibrium electrowetting contact angle, θ, with the applied bias (values are reported vs Pt wire pseudo-reference electrode) at the (a) HOPG–1 M LiClO_4(PC)_ and (b) HOPG–20 m LiTFSI_(aq)_ interfaces in hexadecane. Measurements were conducted under static conditions based on the protocol described in the Experimental Section. Changes in apparent electrowetting contact angle, θ, during wetting/dewetting cycles at the (c) HOPG–1 M LiClO_4(PC)_ and (d) HOPG–20 m LiTFSI_(aq)_ interfaces in hexadecane. One cycle corresponds to two consecutive potential pulses from 0 to +1.1 V vs pseudo-Pt. The changes in θ were extracted by recording the dynamics of the advancing/receding motions using a frame rate of 50 fps (see Movies S4 and S5). Inset: Droplet images at applied biases of 0 and +1.1 V vs pseudo-Pt. Scalebars correspond to 100 μm.

In this section, we aim to explore the applicability of the systems described previously where anion intercalation is used as a tool to drive electrowetting towards the development of biphasic systems with amplified response. The latter strategy is widely applied in various optofluidic technologies to enhance contact angle switching.^11^Figure 7a shows the electrowetting response of a 1 M LiClO_4(PC)_ droplet on HOPG immersed in the non-polar solvent hexadecane (see Figure 1c). The use of hexadecane as the surrounding, insulating phase results in a maximum equilibrium contact angle (ca. 145°) being more than twice the θ_pzc_ at the PC–air interface (ca. 65°). This can be explained by the low liquid–liquid interfacial surface tension, γ_LL_, (i.e., 13.81 ± 0.18 mN m^–1^) at the 1 M LiClO_4(PC)_–hexadecane interface (see Table S2).^84^ For *E* > 0, substantial contact angle changes, even exceeding 120° at the most positive applied potential bias, are observed. Notably, electrowetting is induced with a near-zero voltage threshold, in contrast to some of the most recent EWOD configurations (including commercial technologies) that require an energy input of more than 10 V.^85^ Moreover, previous studies on EWOC systems at the liquid–liquid interface involving aqueous electrolytes^41,43^ report threshold voltage values of more than 1.6 V due to the presence of an ultra-thin insulating layer on the surface of the electrode from the surrounding oil phase (see Table S3). We attribute the threshold voltage elimination in our system to the use of the non-aqueous electrolyte LiClO_4(PC)_ that shows a higher wetting affinity with graphite, as also evidenced by the relatively low work of adhesion (i.e., the reversible thermodynamic work required to separate two immiscible phases^86^) estimated for the 1 M LiClO_4(PC)_–HOPG system in air (see Table S1). This is highlighted in the cyclic voltammogram recorded at the 1 M LiClO_4(PC)_ droplet on HOPG in hexadecane (Figure S4), being almost identical to that recorded at the liquid–air interface (Figure 2b). The similar features in the two voltammograms strongly indicate the unrestricted charge transfer at the interface (i.e., ClO_4_^–^ intercalation), since the presence of an insulating interlayer would significantly increase the required overpotential of the electrochemical processes (see, e.g., the Supporting Information in ref (43).) and hence introduce a threshold voltage region in the electrowetting response. For *E* < 0, in line with what is seen for the 1 M LiClO_4(PC)_–HOPG interface in air (Figure 2a), the contact angle changes are less significant compared to the positive potential region, with changes of up to ca. 55° at the negative potential limit.

The same approach was also applied using the highly concentrated aqueous electrolyte 20 m LiTFSI_(aq)_. Figure 7b shows similar trends to the LiClO_4(PC)_, with contact angle changes of up to ca. 120° at the positive potential limit and smaller contact angle variations in the negative branch of the θ vs *E* plot. The enhancement of the electrowetting response in the highly concentrated aqueous electrolyte used in this study, compared to previous reports using aqueous alkali metal halides,^43,44^ is ascribed to the presence of the large organic TFSI^–^ anions. The latter adsorb strongly on the surface of graphite at *E* > *E*_pzc_ (as indicated by the considerably negative value of *E*_pzc_, i.e., ca. −0.8 V; see Figure 3) and thereby decrease the solid–liquid interfacial tension upon positive polarization, resulting in the observed “more effective” decrease in contact angle in this potential range.

To provide insights into the influence of the intercalation/deintercalation reactions rate on the electrowetting response, we monitored the changes in contact angle under continuous polarization. From the data shown in Figure S5, it can be inferred that intercalation/deintercalation of ClO_4_^–^ is faster compared to the large TFSI^–^ anions that cause larger expansion in the galleries of graphite (see the relevant discussion).

The effect of step edge coverage on the kinetics of the intercalation/deintercalation processes, and hence the electrowetting response within the potential range where the reactions occur, was also investigated. Toward that direction, we monitor the timescales of the droplet’s advancing motion on different grades of HOPG, i.e., ZYA (0.4 ± 0.1°) and ZYB (0.8 ± 0.2°). From the indicative timescales shown in Figure S6 and the corresponding average timescales for each system, we find that intercalation is faster on ZYA HOPG than ZYB by a factor of ca. 16 and 70 for the LiClO_4(PC)_ and LiTFSI_(aq)_ electrolytes, respectively (see discussion in the Supporting Information).

From the applications point of view, reproducibility of electrowetting is of paramount importance for the successful and reliable operation of a device. To examine the reproducibility of the electrowetting response in the developed biphasic systems, we monitored the contact angle changes during 200 consecutive wetting/dewetting cycles (see Experimental Section in the Supporting Information). Figure 7c,d shows the performance of the LiClO_4(PC)_–hexadecane and LiTFSI_(aq)_–hexadecane systems, respectively. The results demonstrate the highly reproducible changes in contact angle over 200 consecutive cycles (see Movies S4 and S5), highlighting the promising character of the reported biphasic systems for applications in electrowetting-based devices.

## Conclusions

We demonstrate that in the presence of anions exhibiting appropriate difference between intercalation and solvation energies, hence being able to intercalate into graphite, the intercalation process governs the electrowetting response of the host material in both aqueous and non-aqueous solvents. Significant contact angle variations of more than 50° are recorded at the liquid–air interface within the potential window of intercalation. The overall mechanism of the phenomenon cannot be explained by the Young–Lippmann equation used to interpret the response of electrowetting-on-dielectric systems, indicating the strong effect of the underlying potential-dependent reactions on electrowetting. The structural changes identified by in situ Raman spectroscopy during intercalation/deintercalation were used to provide insights into the influence of intercalation staging on the rate and reversibility of electrowetting. By tuning the size of the intercalant and the stage of intercalation, the initial graphitic structure can be retained and fully reversible electrowetting becomes feasible, with a significantly enhanced response compared to previously reported purely capacitive systems. Expanding our strategy to the design of biphasic systems, we develop model systems operating on the basis of anion intercalation into graphite with fully reproducible electrowetting responses and unprecedented contact angle changes of up to 120° with applied potentials below 2 V and near-zero threshold voltage. To the best of our knowledge, the performance of these systems is the best reported to date for electrowetting directly on conductors and it even surpasses that of state-of-the-art electrowetting-on-dielectric systems, which exhibit one to two orders of magnitude higher input energy. Our work is expected to provide fundamental insights into the wetting phenomena of graphite in various solvents under the application of an external field, while at the same time it should stimulate further research toward the design of carbon-based dielectric-free electrowetting-based devices.

## Supporting Information Available

Materials and chemicals, preparation of the electrodes, electrowetting setup configuration, PTFE cell configuration, synthesis and NMR spectra (^1^H, ^13^C, ^19^F, and ^7^Li) of EMIM-TFSI, surface tension and mass density measurements, electrochemical measurements, calculation of capacitance from electrochemical impedance measurements, contact angle measurements, in situ Raman spectroscopy measurements, predicting the electrowetting response using the Y–L equation, contact angle variations upon deep intercalation/deintercalation in EMIM-TFSI, surface electrochemistry of LiClO_4(PC)_ in hexadecane, interfacial surface tension and work of adhesion at the liquid–liquid interface, dynamic measurements in the biphasic systems, electrowetting under AC in the biphasic systems, optical images of the droplets at selective potential biases for the systems under study, and electrowetting performance of selected biphasic systems reported in the literature (PDF)
Changes in apparent contact angle during 200 wetting/dewetting cycles for a 1 M LiClO_4(PC)_ droplet on HOPG in air (MP4)
Changes in apparent contact angle at the EMIM-TFSI|HOPG interface in air upon application of a constant potential pulse for 200 s at +1.7 V with subsequent stepping to 0 V (MP4)
Changes in apparent contact angle at the 20 m LiTFSI_(aq)_|HOPG interface in air upon application of a constant potential pulse for 200 s at +0.8 V with subsequent stepping to 0 V (MP4)
Changes in apparent contact angle during 200 wetting/dewetting cycles following the protocol described in the Experimental Section, for a 1 M LiClO_4(PC)_ droplet on HOPG in hexadecane (MP4)
Changes in apparent contact angle during 200 wetting/dewetting cycles following the protocol described in the Experimental Section, for a 20 m LiTFSI_(aq)_ droplet on HOPG in hexadecane (MP4)
