# Supplementary material for: Anion Intercalation into Graphite Drives Surface Wetting
Source: J Am Chem Soc. 2023 Mar 28;145(14):8007–20. doi: 10.1021/jacs.2c13630 (PMC10103168; doi:10.1021/jacs.2c13630)
Supplement: Supplementary file 1 — ja2c13630_si_001.pdf [file ja2c13630_si_001.pdf]

Supporting Information

Anion intercalation into graphite drives surface wetting

Athanasios A. Papaderakis[1,2], Andinet Ejigu[1,2], Jing Yang[1,2], Amr Elgendy[1,2,5], Boya Radha[3,4], Ashok Keerthi[1,3], Anne Juel[3], Robert A. W. Dryfe[1,2*]*

[1]Department of Chemistry, University of Manchester, Oxford Road, Manchester, M13 9PL, United Kingdom

[2]Henry Royce Institute, University of Manchester, Oxford Road, Manchester, M13 9PL, United Kingdom

[3]Department of Physics and Astronomy, University of Manchester, Oxford Road, Manchester, M13 9PL, United Kingdom

[4]National Graphene Institute, University of Manchester, Oxford Road, Manchester, M13 9PL, United Kingdom

[5]Egyptian Petroleum Research Institute, 11727, Cairo, Egypt

Email: athanasios.papaderakis@manchester.ac.uk (A.A.P.), robert.dryfe@manchester.ac.uk (R.A.W.D)

Table of Contents

1. Experimental Section **S3**

1.1 Materials and chemicals S3

1.2 Preparation of the electrodes S3

1.3 Electrowetting setup configuration S3

1.4 PTFE cell setup configuration S4

1.5 Synthesis and NMR of 1-ethyl-3-methylimidazolium bis(trifluoromethanesulfonyl) imide S4

1.6 Surface tension and mass density measurements S6

1.7 Electrochemical measurements S6

1.8 Calculation of capacitance from electrochemical impedance measurements S7

1.9 Contact angle measurements S8

1.10 In-situ Raman spectroscopy measurements S8

2. Results and Discussion Section **S9**

2.1 Predicting the electrowetting response using the Young – Lippmann equation S9

2.2 Contact angle variations upon intercalation/deintercalation in the ionic liquid electrolyte S10

2.3 Surface electrochemistry of $LiClO_{4(PC)}$ in hexadecane S11

2.4 Interfacial surface tension and work of adhesion at the liquid|liquid interface S11

2.5 Dynamic measurements in the biphasic systems S13

2.6 Electrowetting under AC in the biphasic systems S16

2.7 Optical images of the droplets at selective potential biases for the systems under study S17

2.8 Electrowetting on conductors - performance of selected biphasic systems reported in the literature S18

2.9 Captions of movie files S19

3. References **S20**

1. Experimental Section

1.1 Materials and chemicals

HOPG (ZYA and ZYB qualities, mosaic spread 0.4±0.1° and 0.8±0.2°) was purchased from Scanwel, UK. Lithium perchlorate (98 %) from Alfa Aesar, lithium bis(trifluoromethanesulfonyl)imide, (99 %) from Fluorochem, propylene carbonate (anhydrous 99 %) from Alfa Aesar and 1-ethyl-3-methylimidazolium chloride (≥95%) from Sigma were used for the preparation of the electrolytes. Hexadecane (Reagent Plus®, 99 %) from Sigma was used as the insulating phase in the liquid|liquid electrowetting experiments. Ultra-pure water (18.2 MΩ cm resistivity at 25°C, Milli-Q Direct 8) was used in all studies involving aqueous solutions.

1.2 Preparation of the electrodes

HOPG served as the working electrode. Electrical connection was made by stripping an enameled Cu wire (RS components, UK) for about 1 cm at each end and adhering one side to the edge plane of the HOPG with silver conductive epoxy (RS components, UK). After curing for 24 h, the silver epoxy was covered by an insulating resin and left to dry for 3 h. The reference electrode used for the capacitance experiments was a custom-made Ag/AgCl $_{(3M\ KCl)}$ electrode with an agarose gel frit. Its detailed preparation procedure can be found in the supplementary material of [1]. A Pt wire was once again used as a counter electrode. In all cases, prior to each measurement the potential of the reference electrode was recorded with respect to a commercially available Ag/AgCl $_{(3M\ KCl)}$ electrode (from Sigma) in a saturated KCl solution to exclude the possibility of potential drifts among different measurements.

1.3 Electrowetting setup configuration

The setups used for the liquid|air and liquid|liquid electrowetting experiments are displayed in Figure 1b and c, respectively. Micropipettes were fabricated by pulling a borosilicate capillary (inner diameter 0.84 mm, outer diameter 1.5 mm, length 10.16 cm, from World Precision Instruments, UK) with a Sutter P-97 Flaming/Brown Micropipette puller. The inner diameter of the tip in the resultant micropipettes was ca. 5-6 µm. A microinjector (PV820 Pneumatic PicoPump, from World Precision Instruments, FL, US) was used to deposit a droplet on the surface of HOPG by controlled expulsion of the electrolyte. A platinum wire (99.99% purity, 0.05 mm diameter, from Advent, UK), carefully placed on the upper inner part of the micropipette was used as a counter and pseudo-reference electrode. The position of both the HOPG and the micropipette was controlled using manual micro-positioners (Thor Labs). The micropipette was brought close to the surface of the working electrode and the smoothest regions of the HOPG were targeted. In the

case of the liquid|liquid electrowetting experiments, a quartz container was also filled with the surrounding (light) insulating phase. A Photron FASTCAM SA3 high speed camera controlled via Photron FASTCAM Viewer and a Storz Xenon Nova 300 light source were used in static and dynamic (stability tests) experiments. In the case of the stability tests, the frame rate was adjusted to 50 fps.

1.4 PTFE cell setup configuration

For the capacitance experiments the setup used is illustrated in Figure 1d. It consists of a PTFE cylinder (of ca. 0.2 cm^3) with a disk-shaped opening of 3 mm diameter. To ensure that no leakage of the solution occurs, the bottom part of the cylinder was sealed onto the HOPG substrate with a thin (ca. 1 mm) poly(dimethylsiloxane), PDMS, gel layer (Sylgard™ 527, Dow Corning). To set up the cell, a smaller cylinder (3 mm in diameter) was mounted through the disk-shaped opening to seal the Teflon cell and leave an annular region ca. 1 mm deep below the PTFE cylinder where the PDMS mixture was carefully injected. The whole assembly was then transferred to an oven where it was kept at 90°C for 2 h to allow the PTFE gel to cure, followed by cooling at room temperature for at least 3 h and removal of the cylinder.

1.5 Synthesis and NMR of 1-ethyl-3-methylimidazolium bis(trifluoromethylsulfonyl) imide

1-ethyl-3-methylimidazolium chloride (75 g, 0.5115 moles) and lithium bis(trifluoromethanesulfonyl) imide (154 g, 0.54 moles) were dissolved separately in water (100ml) using two different flasks. Subsequently, the solutions were slowly mixed and the resultant mixture was heated at 40 ℃ under stirring overnight. After full mixing, the final solution was added to a separating funnel until two distinct phases formed. The bottom phase, containing the ionic liquid and LiCl impurities, was collected, washed with water under stirring (water to ionic liquid volume ratio greater than two) and transferred again to the separating funnel. The process was repeated for at least 10 times to remove LiCl completely. Finally, the pure (free of LiCl impurities) ionic liquid was heated at 70 ℃ under vacuum (6×10^{-2} mbar) for 3 days.

Figure S1 shows the ^{1}H (Figure S1a) and ^{13}C (Figure S1b) NMR spectra of the synthesized ionic liquid electrolyte. The NMR measurements were recorded using a Bruker Avance II+ 500 MHz NMR spectrometer equipped with a 5 mm Bruker Prodigy Cryo probe. All NMR data were collected at 298 K. For the analysis of the electrolyte, NMR tubes equipped with a coaxial insert filled with a mixture of 10% TMS, 10% TFT and 80% deuterated-DMSO were used in order to lock the magnetic field. The observed ^{1}H and ^{13}C chemical shifts are in line with what is reported in the literature for pure EMIM-TFSI,[2] demonstrating the high purity of the synthesized ionic liquid. Figures S2a and S2b show the recorded ^{19}F-NMR and ^{7}Li-NMR spectra, respectively. The absence of any chemical shift related to ^{7}Li indicates the successful removal of Li^{+} from the ionic liquid. The complete removal of Cl^{-} ions is demonstrated in the absence of any peaks associated with Cl^{-} intercalation and/or chlorine evolution in the CV of Figure 4 in the main text.

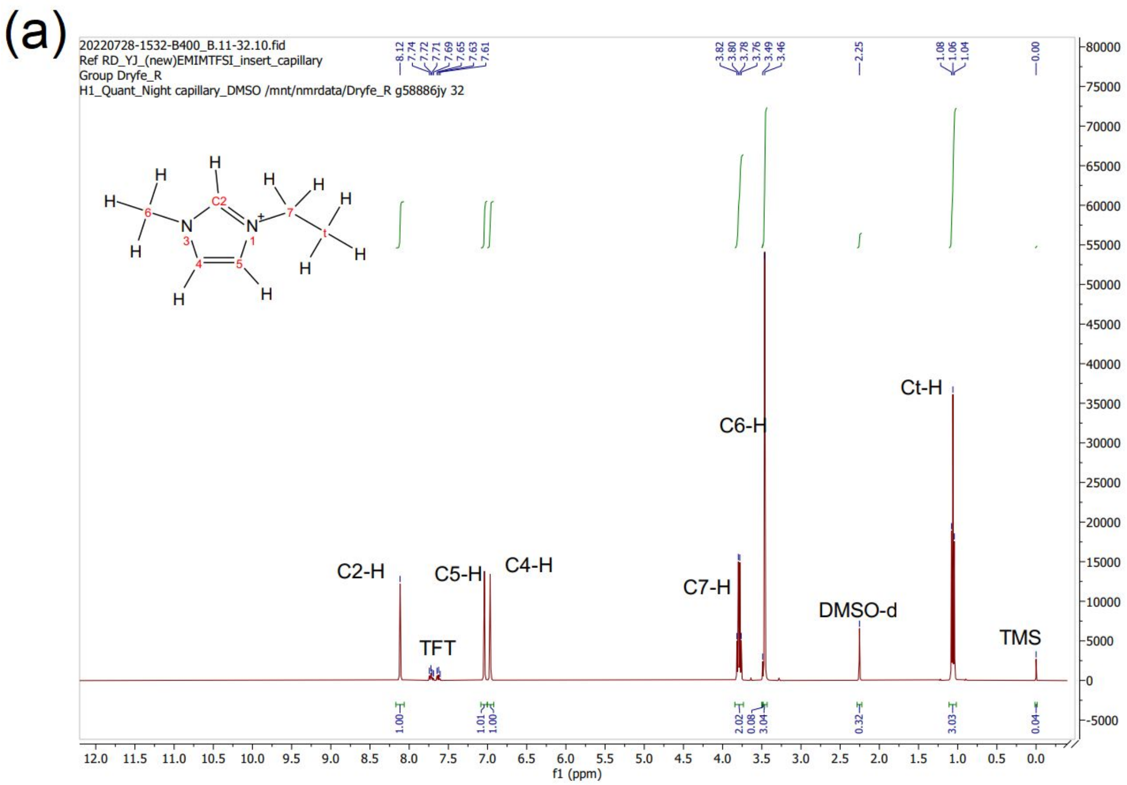

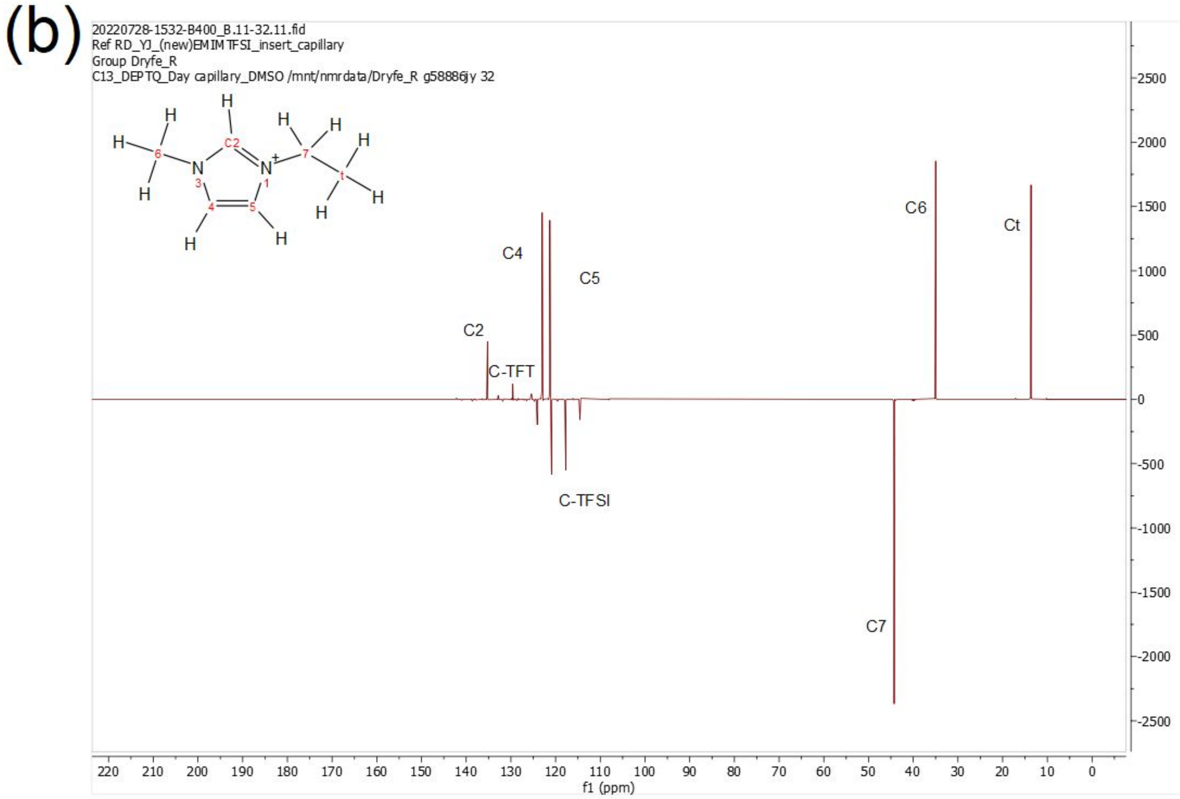

Figure S1: (a) ^{1}H and (b) ^{13}C-NMR spectra of the synthesized EMIM-TFSI ionic liquid electrolyte.

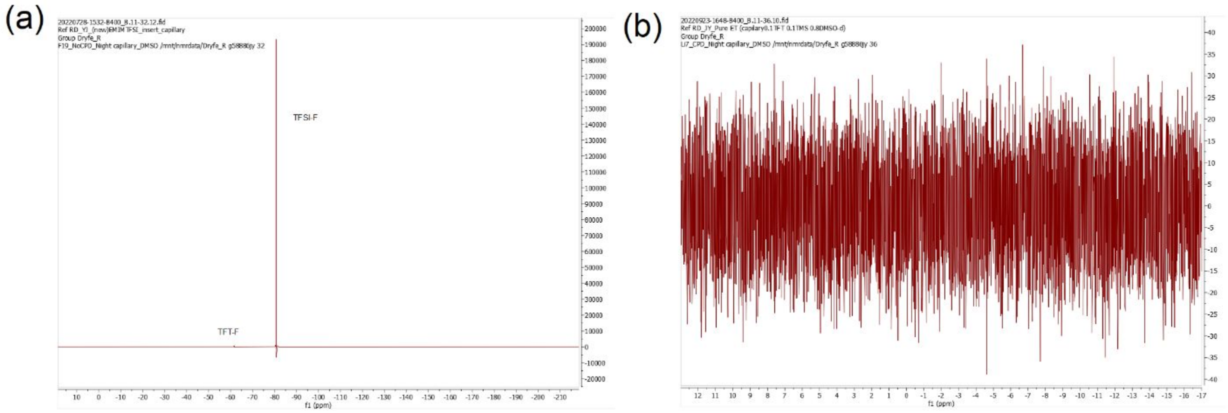

Figure S2: (a) ^{19}F-NMR and (b) ^{7}Li-NMR spectra of the synthesized EMIM-TFSI ionic liquid electrolyte.

1.6 Surface tension and mass density measurements

The liquid-air surface tension, γ_{LV}, of the electrolytes and solvents studied, was determined experimentally at 21°C by implementing the pendant drop method (droplet volume ca. 20 ± 0.5 μL) using a Theta Optical Tensiometer (Biolin Scientific, Sweden) running OneAttension software version 2.3 and applying the Young – Laplace equation. The liquid-liquid surface tension, γ_{LL}, was determined adopting the same approach, using a glass vial as a container of the light phase. The density of the solutions was calculated by weighing (using a 4-digit high precision analytical scale from Ohaus) a constant volume of each solution, i.e., 1000 μL, collected with a calibrated automatic micropipette.

1.7 Electrochemical measurements

All electrochemical experiments were performed on an Autolab PGSTAT302N potentiostat from Metrohm equipped with the FRA32 module and operated with Nova 1.11.2 software. Each measurement was conducted on a freshly cleaved HOPG surface. Prior to the experiment, the Pt wire, which served as a counter and pseudo-reference electrode, was flame cleaned with a blue butane flame. To avoid the contamination of the HOPG by the adsorption of air-bound hydrocarbons, the working solution was deposited on the working electrode within 1 min of cleaving the surface. Unless specified otherwise, the applied potential, E, throughout the main text is referred vs. pseudo-Pt. Potential values recorded vs. Ag/AgCl $_{(3M\ KCl)}$ reference electrode (used in capacitance studies) were converted to the pseudo-Pt potential scale (used in electrowetting experiments), by measuring the potential difference between the two electrodes in each electrolyte used, until equilibrium was reached (i.e., dV/dt < 100 μVs^{-1}). Five different measurements were performed, and the data were averaged to give the final value. The experimental protocol used for the static measurements involved the application of consecutive potential pulses from 0 V to the desired anodic and cathodic potential limit with a step of either 50 or 100 mV. The duration of the pulses was adjusted to 3 s; this time was found to be sufficient to attain equilibrium. A similar strategy was

adopted for the stability tests, in which the potential was directly stepped in between the desired values. The duration of the pulses was 0.5 s. Each repetition represents one cycle.

The AC electrowetting experiments were performed by applying consecutive DC potential pulses between −0.5 and +1.1 V vs. pseudo-Pt with a superimposed sinusoidal perturbation of 10 mV RMS peak-to-peak amplitude, E_0, at various constant frequencies in the range of 100 kHz to 1 Hz. To probe the effect of the imposed voltage amplitude on the electrowetting response the contact angle changes were monitored at a constant applied bias and frequency with varied E_0. The experimental protocol used for the dynamic measurements using different grades of HOPG, i.e., ZYA and ZYB was composed of a potential pulse from 0 to +1.1 V for the 1 M $LiClO_{4(PC)}$|Hexadecane system and from −0.5 to +1.5 V for the 20 m $LiTFSI_{(aq)}$|Hexadecane system. The duration of the pulse was adjusted based on preliminary experiments to attain a steady state response for the electrolyte used, that is an equilibrium value for the contact angle at each applied bias. The frame rate used was chosen to successfully probe the timescales of the droplet's motion within the timeframe of the experiment, i.e., recording at least 30-50 points between the equilibrium plateaus for the wetting states. The characteristic time for the advancing motion for each electrolyte was estimated adopting the following strategy: the mathematical matrix corresponding to each image recorded during the experiment was subtracted by the matrix describing the image of the droplet at equilibrium exhibiting the highest contact angle (i.e., at 0 and -0.5 V for the non-aqueous and aqueous electrolyte respectively), denoted as the reference matrix. Subsequently, the sum of all elements in each of the resultant matrices was calculated and the data were plotted relative to the time of the experiment based on the number of frames and the corresponding frame rate. Finally, the characteristic times used for the determination of the timescales were taken to be 90% of the corresponding average steady state response. The latter is shown in the y-axis of Figure S5 as a dimensionless number with no physical meaning. The overall approach is based on the fact that changes in contact angle as a consequence of the applied bias, will be depicted as contrast differences, i.e., a change in the value of the matrix element corresponding to each pixel (e.g., subtraction of two identical images will result in a zero matrix). The larger the contact angle changes, the larger the contrast differences and hence a higher sum is derived. On this basis, monitoring the changes in the sum of the pixel values in each image matrix can provide us with an estimation of the timescales (in complete analogy with monitoring the contact angle changes, see reference 45). Cyclic voltammetry (CV) experiments were carried out in the microdroplets performed in all cases at a scan rate of 100 mV s^{-1}.

1.8 Calculation of capacitance from electrochemical impedance measurements

Electrochemical impedance spectroscopy (EIS) measurements were performed in the frequency range between 20 kHz – 1 Hz, using an imposed AC rms amplitude of 7 mV peak-to-peak. The EIS experimental data was evaluated for its compliance with Kramers-Kroning (KK) criteria by fitting the AC response of the system to the admittance representation of a theoretical circuit containing a ladder of *n* RC elements in series, with an additional capacitance and/or inductance in parallel to the ladder structure, using the

software developed by Boukamp.[3] The compliance with KK criteria was assured for all data by the values of the relative residuals, calculated to be less than 0.5 % for both the real and imaginary parts of the impedance and the chi-square parameter which was found to be on the order of 10^{-7} for the complete data series. Capacitance was extracted from the EIS data by adopting the graphical approach developed by Orazem and co-workers for systems exhibiting frequency dispersion effects.[4] The value of the constant phase exponent, α, was calculated by performing a linear fit to the plot $logZ_{im}$ vs. $logf$, where Z_{im} and f represent the imaginary part of the total impedance in Ω and the applied frequency in Hz, respectively. The effective capacitance, C_{eff}, was then calculated at each frequency using the following equation:

$$C_{eff} = sin\left(\frac{\alpha\pi}{2}\right)\frac{-1}{Z_{im}(2\pi f)^{\alpha}} \quad (S1)$$

The final capacitance values, C, were determined by averaging the obtained C_{eff} values in the frequency range within which variations smaller than 0.2 μF cm^{-2} were recorded (linear portion of the C_{eff} vs. f plot).

1.9 Contact angle measurements

Contact angle values were extracted from the recorded images of the droplets using a custom-made algorithm written in MATLAB® (MathWorks Inc., Natick, MA, USA). The process initially involved background subtraction by applying the built-in Canny edge detection algorithm. In the next step, the resulted arc (representative of droplet edge) was fitted to a circle equation by means of the incorporated Levenberg-Marquardt non-linear squares fitting algorithm. The contact angle was then determined by using the calculated coefficients of the fitted equation and applying the formula:

$$\begin{cases} \theta = 90^o - \left|arcsin\left(\frac{y_c - y_s}{r}\right)\right| \times \frac{180}{\pi},\ \theta \leq 90^o \\ \theta = 90^o + \left|arcsin\left(\frac{y_s - y_c}{r}\right)\right| \times \frac{180}{\pi}\ ,\ \theta \geq 90^o \end{cases} \quad (S2)$$

where y_c, y_s and r are the y coordinates of the center of the circle, its projection relative to the contact line and the radius of the droplet, respectively. The results were also compared with those extracted using the drop shape analysis plugin[5] (developed by Aurélien Stadler and Daniel Sage) embedded in the ImageJ open-source software[6] (developed by Wayne Rasband).

1.10 In-situ Raman spectroscopy measurements

Raman experiments were performed using a Renishaw inVia microscope with a 532 nm excitation laser operated at a power of 0.274 mW, with a grating of 1800 lines per mm and a 50 × long working distance

objective. The electrochemical cell used for the in-situ measurements was provided by ECC-Opto-Std/El-Cell. It was comprised of a commercial activated carbon (YEC-8B, Fuzhou Yihuan Carbon Co., Ltd.) negative electrode and a freestanding KS4 graphite (Imerys Graphite & Carbon Switzerland Ltd.) as the positive electrode. A glass microfiber membrane soaked with the ionic liquid electrolyte was used as a separator. For the positive electrode, a titanium current collector was used with a small hole in its middle (diameter ca. 1 mm). The positive electrode film was prepared by thoroughly grinding the required amount of KS4 graphite powder with a pestle and mortar. During the process, small amounts of water were added using a microliter pipette, to assist the formation of a homogeneous graphite paste. 10% of polytetrafluoroethylene (80 wt.% dispersion in water from Sigma) was subsequently added in the mixture as binder. The resultant paste was further treated with a pestle to form a thin film (less than 0.5 mm thick) and then oven-dried at 40°C overnight. The exciting laser beam was shone onto the rear of the KS4 electrode through the thin glass window and the small hole in the titanium current collector. Raman measurements were recorded at various voltages, while the cell was subjected to a CV experiment from 1 V to 2.6 V at a scan rate of 10 mV s^{-1}.

2. Results and Discussion

2.1 Predicting the electrowetting response using the Young – Lippmann equation

In EWOD, the changes in the macroscopic contact angle with respect to the applied bias are described by the Young-Lippmann (Y-L) equation; see equation 2 in the main text. In these systems C is practically related to the thickness of the dielectric layer and its relative permittivity and is considered to be constant (i.e., independent of E) for a specific type of material with a defined thickness. Also, the electrochemical interface is considered ideally polarizable, i.e., no faradaic reactions occur. In the absence of a dielectric layer (EWOC), C is governed by the direct interaction of the electrolyte ions with the electrochemically active substrate, e.g., adsorption processes, the organization of the ions close to the surface,[7,8] the thickness of the double layer governed by the electrolyte nature and its ionic strength,[8] diffusion of ions[9] etc. as well as the intrinsic properties of the electrode, e.g. density of states near the Fermi level and the effect of the applied potential on the position of the latter. All these processes are potential dependent making C a function of the applied bias. In this respect we used the experimentally determined capacitance values of Figure 3 to estimate the changes in contact angle with the applied potential. Additionally, γ_{LV} values were also determined experimentally via the pendant drop method (see section 1.5) and the results are presented in Table S1. This approach involved the following steps: *(i)* The difference of the cosines between θ at each applied potential and θ_{pzc}, $\Delta cos\theta$, was calculated by inserting in the Y-L equation the corresponding C, γ_{LV} and E_{pzc} values for the electrolytes studied (blue squares in Figures 1c and 4). *(ii)* We predicted the Y-L-type curve (red lines in Figures 1c and 4) in the whole potential window studied, by fitting a parabolic function of the form $y = a(x - c)^2$ in the estimated electrowetting data from step *(i)*. In this sense, the parameter a in the fitted equation can be considered as a weighted average value of the capacitance measurements within the capacitive region (effective capacitance). Note, that in the case of EMIM-TFSI, due to the absence of a minimum E value in Figure 3c, we estimated E_{pzc} by treating the constant, c, in the above equation as an adjustable parameter.

Table S1: Liquid-air, γ_{LV}, surface tension values determined for the electrolytes and solvents used in the liquid|air electrowetting experiments by applying the pendant drop method (see section 1.5). The droplet volume for the surface tension measurements was ca. 15 ± 0.5 µL. Work of adhesion, W_{SV}, calculated for the solid|liquid interfaces using the Young-Dupré equation (see below). The mass density, ρ, of the solutions is also given.

System	γ_{LV} / mN m^{-1}	W_{SV} / mN m^{-1}	ρ / g cm^{-3}
1 M $LiClO_{4(PC)}$	42.49 (± 0.1)	60.43	1.247
20 m $LiTFSI_{(aq)}$	32.92 (± 0.21)	43	1.723
EMIM-TFSI	34.91 (± 0.39)	51.22	1.52
PC	40.84 (± 0.17)	-	1.2
Water	72.75 (± 0.2)	-	0.99

The work of adhesion, W_{SV}, for the solid|liquid interfaces reported in the Table above, was estimated by using the Young-Dupré equation[10]

$$W_{SV} = \gamma_{LV}(cos\theta + 1) \quad (S1)$$

where γ_{LV} and θ_{pzc} are the interfacial liquid|air surface tension and the equilibrium contact angle at E_{pzc}, respectively.

2.2 Contact angle variations upon intercalation/deintercalation in the ionic liquid electrolyte

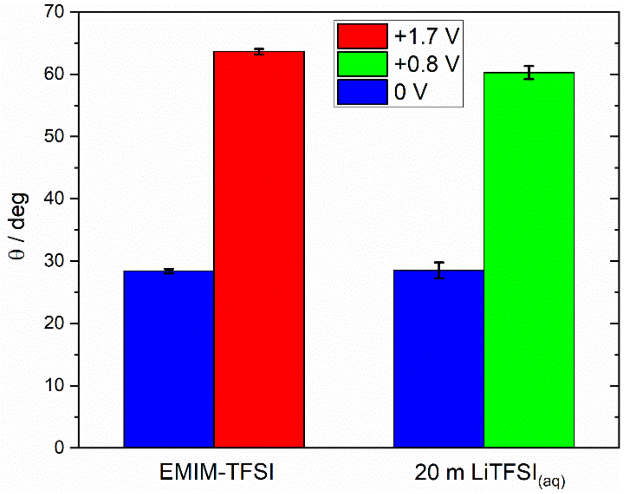

Figure S3: Contact angle variations for EMIM-TFSI and 20 m $LiTFSI_{(aq)}$ in air, after applying a potential pulse at +1.7 V and +0.8 V respectively, for 200 s and subsequently stepping the potential to 0 V. The applied positive potential values correspond to the potential region within which staging intercalation occurs

(see Figure 4, Figure 6 and the relevant discussion in the main text; see also supplementary movies #2 and #3 in the SI). The error bars show the standard deviations for three consecutive cycles.

2.3 Surface electrochemistry of $LiClO_{4(PC)}$ in hexadecane

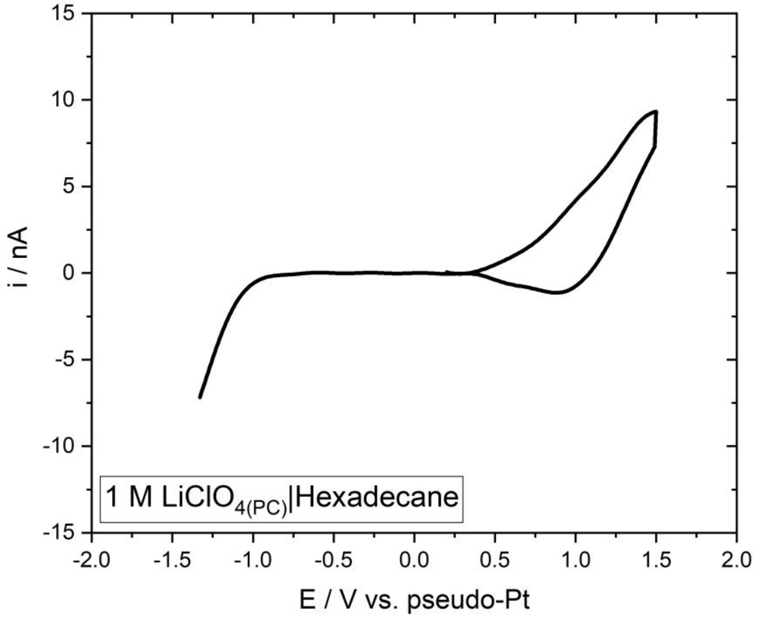

Figure S4: Cyclic voltammogram recorded in a sessile droplet of 1 M $LiClO_{4(PC)}$ deposited on HOPG in hexadecane, using a scan rate of 100 mV s^{-1}.

2.4 Interfacial surface tension and work of adhesion at the liquid|liquid interface

Table S2: Liquid-liquid, γ_{LL}, surface tensions determined for the electrolytes and solvents used in the liquid|liquid electrowetting experiments by applying the pendant drop method (see section 1.5). The droplet volume for the surface tension measurements was ca. 15 ± 0.5 µL. Work of adhesion, W_{LL}, calculated for the liquid|liquid interfaces using the Dupré equation (see below).

System	γ_{LL} / mN m^{-1}	W_{LL} / mN m^{-1}
1 M $LiClO_{4(PC)}$|Hexadecane	13.81 (± 0.18)	50.86
20 m $LiTFSI_{(aq)}$|Hexadecane	14.08 (± 0.16)	41.02
Hexadecane|air	22.18 (± 0.31)	-

The work of adhesion, W_{LL}, for the liquid|liquid interfaces reported in the Table above, was estimated by using the Dupré equation[10]

$$W_{LL} = \gamma_{L1} + \gamma_{L2} - \gamma_{LL} \quad (S2)$$

where γ_{L1}, γ_{L2} are the interfacial liquid|air surface tensions for the liquid 1 and 2 (see Tables S1 and S2) respectively and γ_{LL} the interfacial liquid|liquid surface tension (see Table S2).

2.5 Dynamic measurements in the biphasic systems

Figure S5 presents the contact angle values determined during the forward and reverse scans by sweeping the applied bias from 0 to +1.5 V with varying scan rates. In the case of the 1 M $LiClO_{4(PC)}$|Hexadecane system, a fully reproducible response is observed during both forward and reverse scans, suggesting that the scan rate has a negligible effect on the electrowetting response. Considering that the scan rate used extends up to 1 V s^{-1}, the high degree of reproducibility indicates the fast intercalation/deintercalation kinetics of the ClO_4^- ions into HOPG. On the contrary, for the 20 m $LiTFSI_{(aq)}$|Hexadecane system a clear effect of the scan rate is seen already at the lowest scan rate used, i.e., 100 mV s^{-1}. It is noteworthy that at 1 V s^{-1} the change in contact angle is ca. 52°, i.e., 2.4 times lower than that in the static measurements of Figure 7b, while reversing the scan direction has a negligible effect on the electrowetting response. We ascribe this finding to the surface reconstruction processes occurring during intercalation/deintercalation. It has been already proved that the large $TFSI^-$ anions cause larger lattice expansion in the galleries of graphite and during advanced intercalation stages defects are also introduced (see Figure 6). The higher the extent of the step edges formation/reconstruction, the more energetically demanding the overall process will be and hence slower kinetics are expected. On this basis, the timeframe of the continuous potential pulse applied during the polarization experiments does not provide the time required for the completion of these processes, which is reflected in the observed contact angle changes with scan rate.

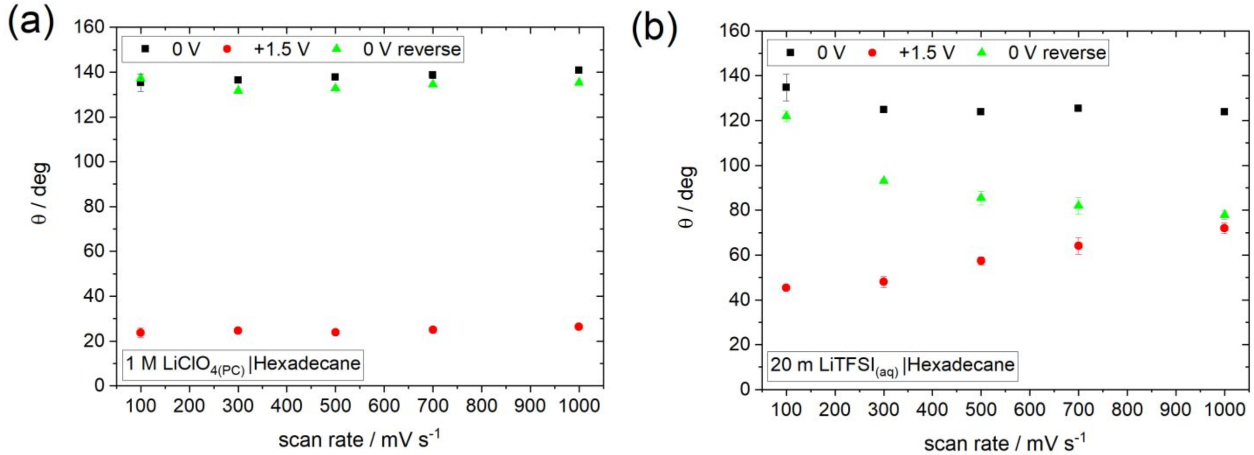

Figure S5: The effect of scan rate and direction on contact angle upon continuous polarization between 0 and +1.5 V vs. Pt pseudo-reference for the (a) 1 M $LiClO_{4(PC)}$|Hexadecane and (b) 20 m $LiTFSI_{(aq)}$|Hexadecane biphasic systems.

Figure S6 shows the indicative timescales of the advancing motions in the two electrolytes studied. From the recorded data it is evident that the characteristic time for the advancing motion of the droplet in both non-aqueous and aqueous electrolytes is significantly lower for the highest-grade quality HOPG. A closer look on the average timescales for each system reveals the estimated characteristic times to be ca. 67.2 $\times 10^{-3}$ ($\pm 11.03 \times 10^{-3}$) and 4.66 ($\pm$ 0.94) s for the ZYA and ZYB HOPG, respectively in 20 m $LiTFSI_{(aq)}$ and ca. 43.7 $\times 10^{-3}$ ($\pm 5.79 \times 10^{-3}$) and 688 $\times 10^{-3}$ ($\pm 79.2 \times 10^{-3}$) s for the ZYA and ZYB HOPG, respectively in 1 M $LiClO_{4(PC)}$. This very interesting observation strongly demonstrates that the kinetics of the intercalation process in both electrolytes are facilitated with decreasing the density of step edges on the surface of HOPG. We interpret this finding based on the energy needed to overcome the van der Waals forces holding together the graphene layers on graphite, in order for intercalation to occur.[11,12] In more detail, the higher number of stacked graphene layers on the larger step edges of the ZYB sample increases the binding forces between the adjacent graphene sheets compared to the ZYA HOPG[13] and therefore more energy is required for the intercalation to commence and subsequently proceed. This is further supported by the observed contact angle changes for the ZYB sample. As can be seen from the insets in Figure S6 b, d the recorded contact angles at the most positive applied bias are higher by a factor of ca. 1.4 and 5.5 for the $LiClO_{4(PC)}$ and $LiTFSI_{(aq)}$ electrolytes respectively, (the equilibrium contact angles are ca. 41° at +1.1 V and 109° at +1.5 V for the $LiClO_{4(PC)}$ and $LiTFSI_{(aq)}$ electrolytes, respectively) compared to those determined for the ZYA sample (see Figure 7). The suppression of electrowetting for lower grades of HOPG is in line with what is reported in the literature for aqueous solutions of $NaClO_4$. [14] Furthermore, the shorter timescale for the $LiClO_{4(PC)}$ electrolyte relative to $LiTFSI_{(aq)}$ (being more profound as the density of step edges increases, i.e., in the ZYB sample) possibly indicates the lower solvation energy of the ClO_4^- anions in PC compared to that of the $TFSI^-$ anions in water. This results in the appropriate difference between the intercalation and solvation energies that facilitates the intercalation of ClO_4^- anions into graphite[15] and therefore promotes electrowetting (a phenomenon being also evident in the significantly smaller changes of the contact angle at the most positive bias between the ZYA and ZYB samples in the non-aqueous electrolyte). The short-term (ca. 10 cycles) reversibility of the phenomenon at the ZYB samples was also explored (experiments not shown) where it was found that despite the damping of the electrowetting response the overall process appears to be reversible.

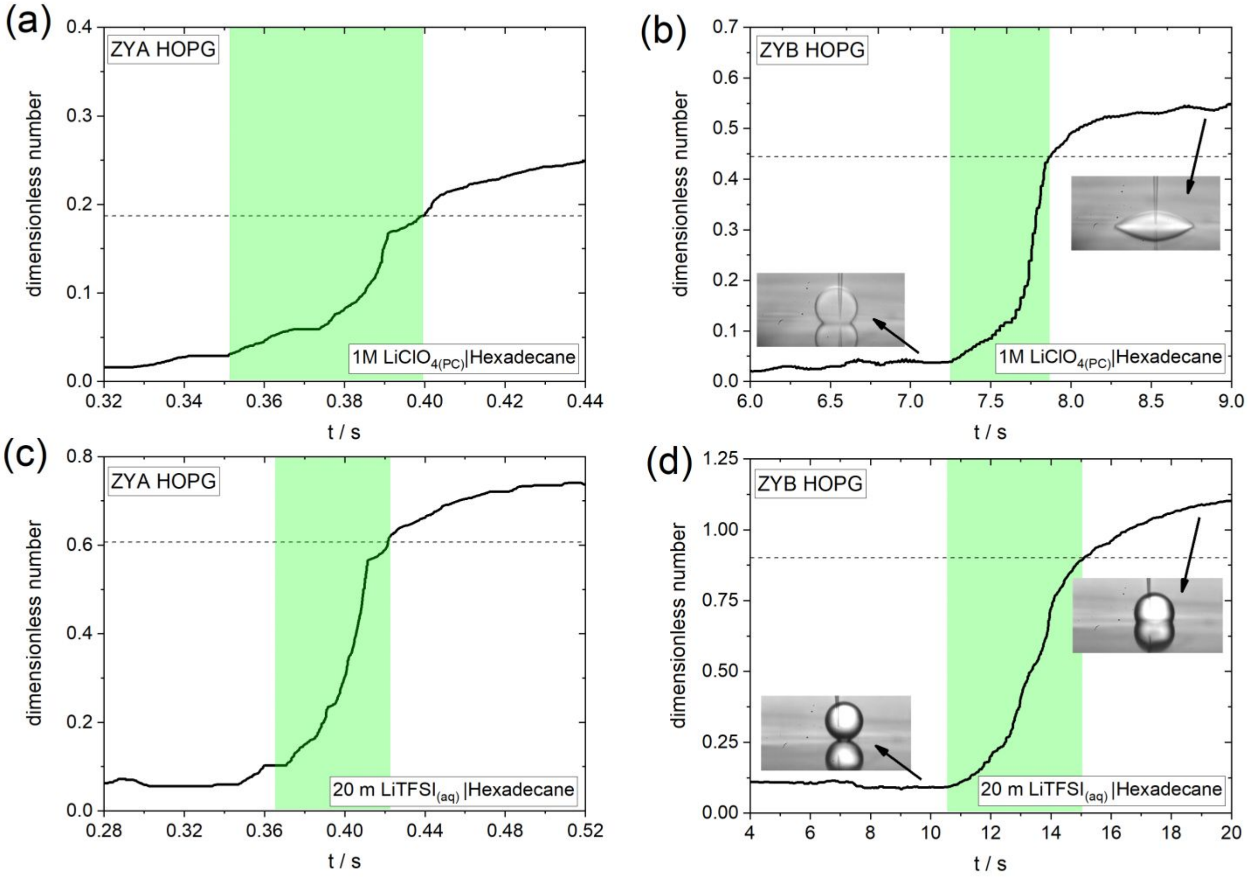

Figure S6: Indicative timescales (highlighted regions) of droplet's advancing motion, recorded for the biphasic systems presented in Figure 7 using as a substrate HOPG of (a, c) ZYA (0.4 ± 0.2^o) and (b, d) ZYB (0.8 ± 0.2^o) grade quality. The potential was stepped from 0 to +1.1 V vs. Pt pseudo-reference for the 1 M $LiClO_{4(PC)}$|Hexadecane system and from -0.5 to +1.5 V vs. Pt pseudo-reference for the 20 m $LiTFSI_{(aq)}$|Hexadecane system (for details about the experimental protocol see the Experimental Section). The extracted timescales are found to be ca. 67.2 $\times$ 10^{-3} (± 11.03 $\times$ 10^{-3}) and 4.66 (± 0.94) s for the ZYA and ZYB HOPG, respectively in 20 m $LiTFSI_{(aq)}$ and ca. 43.7 $\times$ 10^{-3} (± 5.79 $\times$ 10^{-3}) and 688 $\times$ 10^{-3} (± 79.2 $\times$ 10^{-3}) s for the ZYA and ZYB HOPG, respectively in 1 M $LiClO_{4(PC)}$. The derived average values were determined based on three different measurements. Insets (b, d): Droplet images on the ZYB HOPG.

2.6 Electrowetting under AC in the biphasic systems

The electrowetting response of the biphasic systems was also investigated under AC conditions based on the experimental protocol described in the SI. Figure S7a shows the dependence of the apparent equilibrium contact angle, θ, at the HOPG|20 m $LiTFSI_{(aq)}$ interface in hexadecane on the applied AC voltage. From the recorded response it is evident that electrowetting occurs under AC bias, however the overall response is very close to the DC case. Interestingly, no shape oscillations of the droplet arising by the frequency variations on the applied AC voltage pulses were observed, which is in contrast to what is well-established for EWOD systems.[16] Furthermore, as can be seen in Figure S7b, there is no influence of the imposed voltage amplitude, E_0, on the recorded θ. A possible explanation for these findings is the strong effect of the applied potential on the electrode|electrolyte interfacial surface tension due to the occurrence of the underlying electrochemical reactions (in this case anion adsorption/intercalation). On this basis, the overall mechanism of the phenomenon is predominantly of electrochemical nature and hence any electromechanical contributions, e.g., the effect of Maxwell stress on the three-phase contact line, are expected to be less significant. An additional important factor needs to be considered is that based on the instrumentation used, the AC perturbation was superimposed to a constant DC voltage pulse, i.e., DC current flows through the cell. In other words, the faradaic processes, being responsible for the electrowetting response, still occur in a similar way to the static measurements of Figure 7.

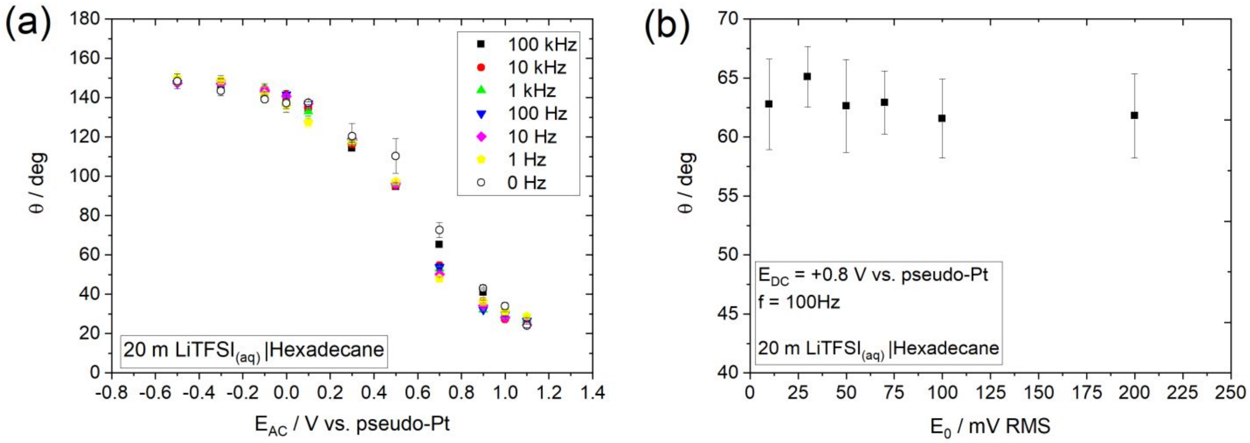

Figure S7: (a) Change in apparent equilibrium electrowetting contact angle, θ, with the applied AC bias, E_{AC}, (values are reported vs. Pt wire pseudo-reference electrode) at the HOPG|1 M $LiClO_{4(PC)}$ interface in hexadecane. Measurements were conducted under static conditions at several constant applied frequencies based on the protocol described in the Experimental Section. The form of the voltage perturbation was sinusoidal with an applied voltage amplitude, E_0, of 10 mV RMS peak-to-peak. (b) The effect of E_0 on θ at a constant applied AC bias and frequency. The former was chosen such to lie within the potential region where anion intercalation occurs (see Figures 4, 7 in the main text and the relevant discussion therein).

2.7 Optical images of the droplets at selective potential biases for the systems under study

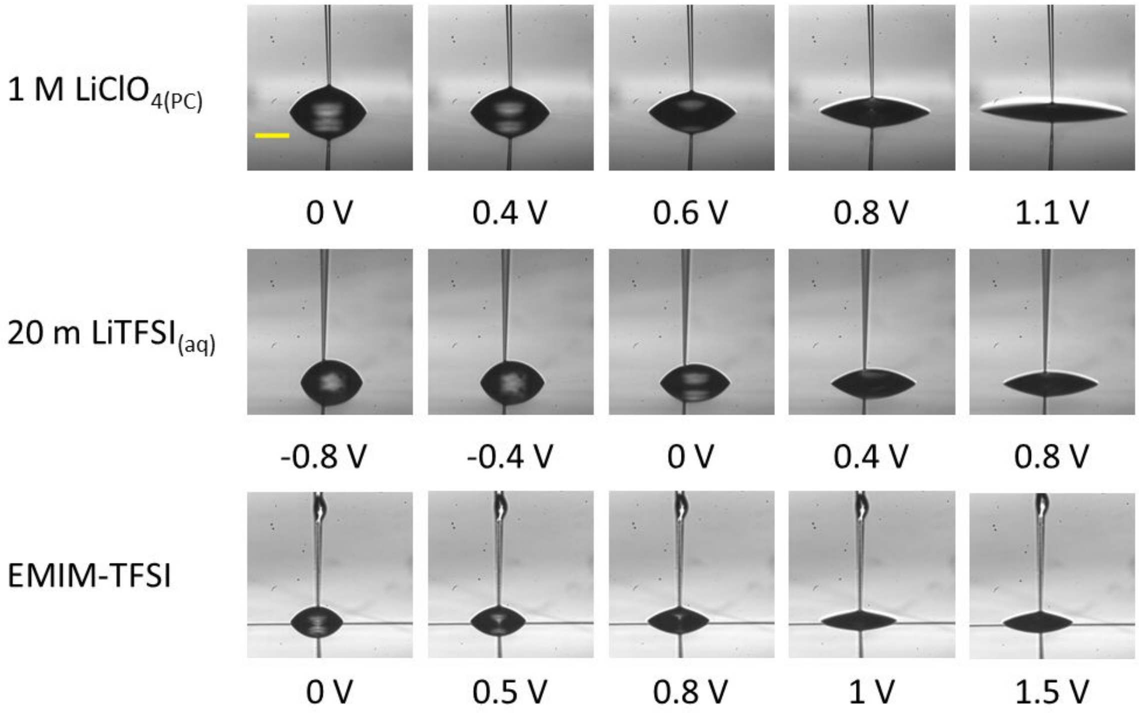

Figure S8: Droplet pictures at indicative potentials within the positive potential window of the data presented in Figures 2 and 4 in the main text; liquid|air electrowetting. Potentials are quoted vs. pseudo-Pt. Scalebar corresponds to ca. 100 μm.

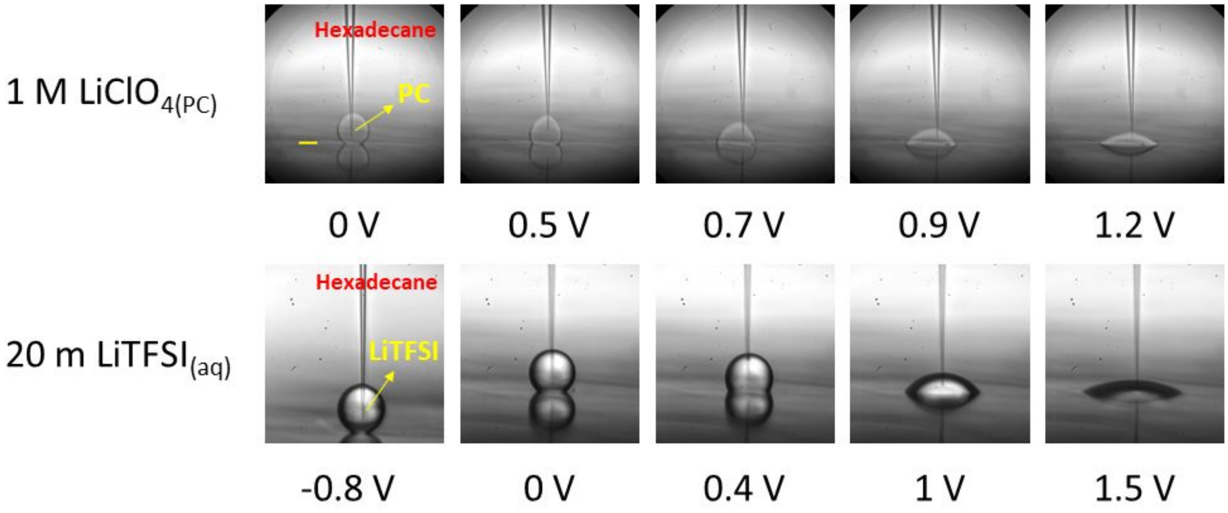

Figure S9: Droplet pictures at indicative potentials within the positive potential window of the data presented in Figure 7 in the main text; liquid|liquid electrowetting. Potentials are quoted vs. pseudo-Pt. Scalebar corresponds to ca. 100 μm.

2.8 Electrowetting on conductors – performance of selected biphasic systems reported in the literature

Table S3: Electrowetting performance of selected systems in the literature (for number of references refer to the main text or SI).

Biphasic system	Electrode	Operational voltage window (V)	Threshold voltage (V)	Maximum contact angle change (deg)	Contact angle hysteresis	Reference
0.5 M $LiCl_{(aq)}$ and 0.01 TBATPB in nitrobenzene	Au	0.9	0.4	30	Yes (CV)/No (potential pulses)	41 (main text)
Novec 7100 in 0.05 M $KClO_{4(aq)}$	Au(111)	1.5	0.1	50	Yes (CV)	85 (main text)
Hexadecane in 0.05 M $KClO_{4(aq)}$	Au(111)	1.3	0.4	40	Yes (CV)	Morooka, et al. [17]
Hexadecane in (0.05 M $KClO_4$ + 2 mM KBr)$_{(aq)}$	Au(111)	1.3	0.4	28	Yes (CV)	Morooka et al. [17]
6 M $LiCl_{(aq)}$ in Hexadecane	HOPG	6	1.6	110	-	43 (main text)
Perfluorodecalin in 10 m $KF_{(aq)}$	HOPG	2.9	0.8	42	No (potential pulses)	45 (main text)
(1 M $LiClO_4$ in propylene carbonate) in Hexadecane	HOPG	3	< 0.05	120	No (potential pulses)	This work
20 m $LiTFSI_{(aq)}$ in Hexadecane	HOPG	3	< 0.2	120	No (potential pulses)	This work

2.9 Captions of movie files

Movie 1: Changes in apparent contact angle, θ, during 200 wetting/dewetting cycles following the protocol described in the Experimental Section, for a 1 M $LiClO_{4(PC)}$ droplet on HOPG in air (see Figure 2d). One cycle corresponds to two consecutive potential pulses from 0 to +1.1 V vs. pseudo-Pt. The playback speed is set to 125 fps, i.e., five times faster than that in the actual recording (50 fps).

Movie 2: Changes in apparent contact angle, θ, at the EMIM-TFSI|HOPG interface in air upon application of a constant potential pulse for 200 s at +1.7 V (i.e., within the potential region where staging intercalation occurs; see Figure 4 and Figure 6 in the main text) with subsequent stepping to 0 V (where deintercalation occurs rapidly; see Figure 4 and Figure 6 in the main text).

Movie 3: Changes in apparent contact angle, θ, at the 20 m $LiTFSI_{(aq)}$|HOPG interface in air upon application of a constant potential pulse for 200 s at +0.8 V (i.e., within the potential region where staging intercalation occurs; see Figure 4 in the main text) with subsequent stepping to 0 V (where deintercalation occurs rapidly; see Figure 4 in the main text).

Movie 4: Changes in apparent contact angle, θ, during 200 wetting/dewetting cycles following the protocol described in the Experimental Section, for a 1 M $LiClO_{4(PC)}$ droplet on HOPG in hexadecane (see Figure 7c). One cycle corresponds to two consecutive potential pulses from 0 to +1.1 V vs. pseudo-Pt. The playback speed is set to 125 fps, i.e., five times faster than that in the actual recording (50 fps).

Movie 5: Changes in apparent contact angle, θ, during 200 wetting/dewetting cycles following the protocol described in the Experimental Section, for a 20 m $LiTFSI_{(aq)}$ droplet on HOPG in hexadecane (see Figure 7d). One cycle corresponds to two consecutive potential pulses from 0 to +1.1 V vs. pseudo-Pt. The playback speed is set to 125 fps, i.e., five times faster than that in the actual recording (50 fps).

3. References

(1) Iamprasertkun, P.; Ejigu, A.; Dryfe, R. A. W. Understanding the Electrochemistry of "Water-in-Salt" Electrolytes: Basal Plane Highly Ordered Pyrolytic Graphite as a Model System. *Chem. Sci.* **2020**, *11* (27), 6978–6989. https://doi.org/10.1039/D0SC01754J.

(2) D'Agostino, C.; Mantle, M. D.; Mullan, C. L.; Hardacre, C.; Gladden, L. F. Diffusion, Ion Pairing and Aggregation in 1-Ethyl-3-Methylimidazolium-Based Ionic Liquids Studied by 1H and 19F PFG NMR: Effect of Temperature, Anion and Glucose Dissolution. *ChemPhysChem* **2018**, *19* (9), 1081–1088. https://doi.org/10.1002/cphc.201701354.

(3) Lasia, A. Conditions for Obtaining Good Impedances. In *Electrochemical Impedance Spectroscopy and its Applications*; Lasia, A., Ed.; Springer: New York, NY, 2014; pp 271–300. https://doi.org/10.1007/978-1-4614-8933-7_13.

(4) Orazem, M. E.; Pébère, N.; Tribollet, B. Enhanced Graphical Representation of Electrochemical Impedance Data. *J. Electrochem. Soc.* **2006**, *153* (4), B129. https://doi.org/10.1149/1.2168377.

(5) *BIG>Drop Analysis*. http://bigwww.epfl.ch/demo/dropanalysis/.

(6) *ImageJ*. https://imagej.nih.gov/ij/index.html.

(7) Williams, C. D.; Dix, J.; Troisi, A.; Carbone, P. Effective Polarization in Pairwise Potentials at the Graphene–Electrolyte Interface. *J. Phys. Chem. Lett.* **2017**, *8* (3), 703–708. https://doi.org/10.1021/acs.jpclett.6b02783.

(8) Brown, M. A.; Goel, A.; Abbas, Z. Effect of Electrolyte Concentration on the Stern Layer Thickness at a Charged Interface. *Angewandte Chemie* **2016**, *128* (11), 3854–3858. https://doi.org/10.1002/ange.201512025.

(9) Pletcher, D. *A First Course in Electrode Processes*, 2nd ed.; RSC publishing: Cambridge, UK, 2009.

(10) Zhang, J. Work of Adhesion and Work of Cohesion. In *Encyclopedia of Tribology*; Wang, Q. J., Chung, Y.-W., Eds.; Springer US: Boston, MA, 2013; pp 4127–4132. https://doi.org/10.1007/978-0-387-92897-5_451.

(11) Schnyder, B.; Alliata, D.; Kötz, R.; Siegenthaler, H. Electrochemical Intercalation of Perchlorate Ions in HOPG: An SFM/LFM and XPS Study. *Applied Surface Science* **2001**, *173* (3), 221–232. https://doi.org/10.1016/S0169-4332(00)00902-8.

(12) Alliata, D.; Kötz, R.; Haas, O.; Siegenthaler, H. In Situ AFM Study of Interlayer Spacing during Anion Intercalation into HOPG in Aqueous Electrolyte. *Langmuir* **1999**, *15* (24), 8483–8489. https://doi.org/10.1021/la990402o.

(13) Shih, C.-J.; Strano, M. S.; Blankschtein, D. Wetting Translucency of Graphene. *Nature Mater* **2013**, *12* (10), 866–869. https://doi.org/10.1038/nmat3760.

(14) Zhang, G.; Walker, M.; Unwin, P. R. Low-Voltage Voltammetric Electrowetting of Graphite Surfaces by Ion Intercalation/Deintercalation. *Langmuir* **2016**, *32* (30), 7476–7484. https://doi.org/10.1021/acs.langmuir.6b01506.

(15) Huang, Z.; Hou, Y.; Wang, T.; Zhao, Y.; Liang, G.; Li, X.; Guo, Y.; Yang, Q.; Chen, Z.; Li, Q.; Ma, L.; Fan, J.; Zhi, C. Manipulating Anion Intercalation Enables a High-Voltage Aqueous Dual Ion Battery. *Nat Commun* **2021**, *12* (1), 3106. https://doi.org/10.1038/s41467-021-23369-5.

(16) Oh, J. M.; Ko, S. H.; Kang, K. H. Shape Oscillation of a Drop in Ac Electrowetting. *Langmuir* **2008**, *24* (15), 8379–8386. https://doi.org/10.1021/la8007359.

(17) Morooka, T.; Tahara, H.; Sagara, T. Effect of Bromide Adsorption on Electrowetting of Au Electrode with Hexadecane. *Electrochimica Acta* **2017**, *251*, 355–362. https://doi.org/10.1016/j.electacta.2017.08.133.
